# The COVIRL-001 Trial: A multicentre, prospective, randomised trial comparing standard of care (SOC) alone, SOC plus hydroxychloroquine monotherapy or SOC plus a combination of hydroxychloroquine and azithromycin in the treatment of non- critical, SARS-CoV-2 PCR-positive population not requiring immediate resuscitation or ventilation but who have evidence of clinical decline: A structured summary of a study protocol for a randomised controlled trial

**DOI:** 10.1186/s13063-020-04407-x

**Published:** 2020-05-25

**Authors:** Eoin Feeney, Deborah Wallace, Aoife Cotter, Willard Tinago, Cormac McCarthy, David Keane, Rabia Hussain, Elena Alvarez Barco, Peter Doran, Patrick Mallon

**Affiliations:** 1 St Vincent’s University Hospital, Dublin, Ireland; 2 School of Medicine, University College Dublin, Dublin, Ireland; 3 Mater Misericordiae University Hospital, Dublin, Ireland; 4 HRB-Trial Methodology Research Network, Galway, Ireland

## Abstract

### Objectives

Hydroxychloroquine has attracted considerable interest as a potential therapy for COVID-19 infection due to its immunomodulatory effects and reported efficiency in clearing upper airways of the virus. Recent data have also suggested the treatment of COVID-19 patients with hydroxychloroquine and azithromycin to cure the associated infection. The objective of this trial is to assess the efficacy of standard of care (SOC) plus hydroxychloroquine monotherapy or SOC plus a combination of hydroxychloroquine and azithromycin in subjects with non-critical, SARS-CoV-2 PCR-positive infection not requiring immediate resuscitation or ventilation but who have evidence of clinical decline compared to SOC alone.

### Trial design

This is a multi-centre, prospective, randomised, open label, 3 arm (ratio 1:1:1) trial with parallel group design.

### Participants

Patients with confirmed diagnosis of COVID-19 (PCR positive) who are non-critical but show evidence of progressive clinical decline at St. Vincent’s University Hospital and Mater Misericordiae University Hospital, Dublin, Ireland. *Inclusion Criteria* Documented COVID-19 positive (positive PCR test on a respiratory sample for SARS-CoV2), Aged ≥18 years male or female. Evidence of progressive clinical decline as defined by: elevated and /or rising (over a 24hour period) inflammatory markers (at least two of in CRP, d-dimer, LDH and/or ferritin above the upper limit of normal), presence of or progression of pulmonary infiltrates on CXR (as decided by the treating physician), new hypoxia requiring >2l/min/28% FiO2 to maintain oxygen saturations ≥94% (or 88-92% in patients with chronic hypercapnic respiratory failure).

### Intervention and comparator

Interventions for participants in this trial are SOC plus hydroxychloroquine monotherapy or SOC plus a combination of hydroxychloroquine and azithromycin. Following randomization, subjects will receive either (Arm 2) SOC plus hydroxychloroquine (400mg BID on day 1 then 200mg twice daily from day 2 to 10) or (Arm 3) SOC plus hydroxychloroquine (400mg BID on day 1 then 200mg twice daily from days 2 to 10) and azithromycin (500mg day 1 and 250mg daily from days 2 to 5). The comparator in this trial is SOC alone (Arm 1).

### Main outcomes

The primary endpoint for this trial is a composite endpoint for time to progression to intubation, non-invasive ventilation, use of immunomodulatory therapy* for COVID-19 infection or death.

*Immunomodulatory therapy refers to use of high dose corticosteroids (methylprednisolone) or new initiation of any humanised monoclonal antibody or convalescent serum

### Randomisation

Eligible patients (351) will be randomised using a central register in the ratio 1:1:1, (117 per arm) with the use of permuted blocks of random sizes. To ensure concealment, the block sizes will not be disclosed. Randomisation will be performed through an interactive web-based electronic data capturing database.

### Blinding

This study is open label. The study will not be blinded to investigators, subjects, or medical or nursing staff. The trial statistician will be blinded for data analysis and will be kept unaware of treatment group assignments. To facilitate this, the randomisation schedule will be drawn up by an independent statistician. Furthermore, we defined objective criteria for the primary outcome to minimize potential bias.

### Numbers to be randomised

A total of 351 patients will be randomised; 117 participants into SOC plus hydroxychloroquine monotherapy, 117 into SOC plus a combination of hydroxychloroquine and azithromycin intervention group and 117 into SOC.

### Trial Status

The COVIRL001 trial (Protocol version 1.4, 05 May 2020) will commence in May 2020 at St. Vincent’s University Hospital and Mater Misericordiae University Hospital, Dublin, Ireland. Recruitment is proceeding with the aim to achieve the target sample size on or before October 2020.

### Trial registration

The COVIRL001 trial is registered on 06 May 2020 under EudraCT number: 2020-001265-36 (https://www.clinicaltrialsregister.eu/ctr-search/search?query=covid-19+AND+University+College+Dublin) and Protocol identification: UCDCRC/20/01.

### Full protocol

The full protocol for COVIRL001 is attached as an additional file, accessible from the Trials website (Additional file 1). In the interest in expediting dissemination of this material, the familiar formatting has been eliminated; this Letter serves as a summary of the key elements of the full protocol (Protocol version 1.4, 05 May 2020). The study protocol has been reported in accordance with the Standard Protocol Items: Recommendations for Clinical Interventional Trials (SPIRIT) guidelines (Additional file 2).

## Keywords

COVID-19; Randomised controlled trial; protocol; hydroxychloroquine; azithromycin

## Supplementary information

**Additional file 1.** COVIRL-001_Protocol_V1.4.

**Additional file 2.** SPIRIT_COVIRL-001.

**Additional file 3.** COVIRL_PIL.

## Data Availability

Individual requests for access to the trial database will be considered in discussion with the local Research Ethics Committee.

