# Supplementary material for: The COVIRL-001 Trial: A multicentre, prospective, randomised trial comparing standard of care (SOC) alone, SOC plus hydroxychloroquine monotherapy or SOC plus a combination of hydroxychloroquine and azithromycin in the treatment of non- critical, SARS-CoV-2 PCR-positive population not requiring immediate resuscitation or ventilation but who have evidence of clinical decline: A structured summary of a study protocol for a randomised controlled trial
Source: Trials. 2020 May 25;21:430. doi: 10.1186/s13063-020-04407-x (PMC7247433; doi:10.1186/s13063-020-04407-x)
Supplement: Supplementary file 1 — Additional file 1. COVIRL-001_Protocol_V1.4. [file 13063_2020_4407_MOESM1_ESM.pdf]

COVID-19 Pre-ICU Trials Ireland

Treatment Study 001

COVIRL-001 - A multicentre, prospective, randomised trial comparing standard of care (SOC) alone, SOC plus hydroxychloroquine monotherapy or SOC plus a combination of hydroxychloroquine and azithromycin in the treatment of non-critical, SARS-CoV-2 PCR-positive population not requiring immediate resuscitation or ventilation but who have evidence of clinical decline.

**Protocol date:**	5th May 2020
**Protocol version:**	1.4
**EudraCT number:**	2020-001265-36
**Sponsor:**	University College Dublin
**Sponsor's Protocol Code:**	UCDCRC/20/01

**Chief Investigator:**	Prof. Patrick Mallon<br>Professor of Microbial Diseases<br>Centre for Experimental Pathogen Host Research<br>UCD School of Medicine<br>Consultant Infectious Diseases Physician<br>St Vincent's University Hospital<br>Dublin 4, Dublin, Ireland
**Site Investigator (s):**	Dr Eoin Feeney<br>Consultant Infectious Diseases Physician<br>St Vincent's University Hospital<br>Dublin 4, Dublin, Ireland<br><br>A/Prof. Aoife Cotter<br>Consultant Infectious Diseases Physician<br>Mater Misericordiae University Hospital<br>Dublin 7, Dublin, Ireland

Co-investigators: see http://www.ucd.ie/medicine/cephr/

PROTOCOL SIGNATURE PAGE

Protocol Title: COVIRL-001 - A multicentre, prospective, randomised trial comparing standard of care (SOC) alone, SOC plus hydroxychloroquine monotherapy or SOC plus a combination of hydroxychloroquine and azithromycin in the treatment of non-critical, SARS-CoV-2 PCR-positive population not requiring immediate resuscitation or ventilation but who have evidence of progressive clinical decline.

Protocol Number: UCDCRC/20/01

Protocol Version/ Date: Version 1.4, 05-May-2020

Sponsor Name: University College Dublin

I, the undersigned, am responsible for the conduct of the trial at this site and agree to the following:

- I understand and will conduct the trial according to the protocol, any approved protocol amendments, ICH GCP and all applicable regulatory authority requirements and national laws.
- I will not deviate from the protocol without prior written permission from the Sponsor and prior review and written approval from Independent Ethics Committee, except where necessary to prevent any immediate danger to the subject.
- I have read and understand fully the SmPCs for the investigational medicinal products; and I am familiar with the Investigational Medicinal Product(s) (IMP) and its use according to this protocol.
- I have sufficient time to properly conduct and complete the trial within the agreed trial period, and I have available an adequate number of qualified staff and adequate facilities for the foreseen duration of the trial to conduct the trial properly and safely.
- I will ensure that any staff at my site(s) who are involved in the trial conduct are adequately trained regarding the Investigational Medicinal Products, the protocol and their responsibilities. In the case of delegating any of my trial responsibilities I will provide the Sponsor with a Delegation of Activities certificate.

	Name	Signature	Date
**SPONSOR**	**Prof Peter Doran**		05-May-2020
**CHIEF INVESTIGATOR**	**Prof Patrick Mallon**		05-May-2020
**SITE PRINCIPAL INVESTIGATOR**			

Document History

Document	Date of Issue	Summary of Change
Version 1.0 - Original protocol	07-Apr-2020	Not applicable
Version 1.1 – Amended protocol	14-Apr-2020	Changes made to Protocol following feedback from the HPRA:<br>- Assessments of Safety section - Amended to include assessment of new neuropsychological AEs.<br>- Exclusion Criteria – based on requests from HPRA on additional considerations.<br>- Sample size calculation
Version 1.2 – Amended protocol	18-Apr-2020	Changes made following Ethics Committee review of the Protocol:<br>- DSMB – Further detail provided on the assignment and functionality of the DSMB for the trial.<br>- Quality Control & Quality Assurance procedures – Monitoring and minimum source data verification procedures defined.
Version 1.3 – Amended protocol	28-Apr-2020	Changes made following HPRA review:<br>- Statistical analysis modified and sample size adjusted accordingly<br>- Additional ECG monitoring to include daily ECG from D1-5 and day 2 ECG to be completed 2-3 hours post dose.
Version 1.4 – Amended protocol	05-May-2020	Changes made following HPRA review:<br>- Clarification that statistician involved in DSMB<br>- Modification to exclusion criteria<br>- Modification to DSMB section

Abbreviations

AE	Adverse Event
AST	Aspartate aminotransferase
ALT	Alanine transaminase
BID	Twice daily
CRF	Case report form
CRP	C-reactive protein
CXR	Chest X-ray
CDAD	Clostridium difficile-associated diarrhoea
DSMB	Data Safety Monitoring Board
ECG	Electrocardiogram
EDTA	Ethylenediaminetetraacetic acid
G6PD	Glucose-6-phosphate dehydrogenase
GCP	Good Clinical Practice
HCQ	Hydroxychloroquine
HPRA	Health Products Regulatory Authority
HRCDC	Health Research Consent Declaration Committee
ICU	Intensive Care Unit
IEC	Independent Ethics Committee
IMP	Investigational Medicinal Product
IRB	Independent Review Board
LDH	Lactate dehydrogenase
PIS	Patient Information Statement
PCR	Polymerase chain reaction
SAE	Serious Adverse Event
SAR	Serious Adverse Reaction
SOP	Standard operating procedure
SPC/SmPC	Summary of Product Characteristics
SUSAR	Suspected Unexpected Serious Adverse Reaction

Protocol Synopsis

Full Title	COVIRL-001- A multicentre, prospective, randomised trial comparing standard of care (SOC) alone, SOC plus hydroxychloroquine monotherapy or SOC plus a combination of hydroxychloroquine and azithromycin in the treatment of non-critical, SARS-CoV-2 PCR-positive population not requiring immediate resuscitation or ventilation but who have evidence of clinical decline.
Short Title	COVID-19 Pre-ICU Trials Ireland - Treatment Study 001 (COVIRL-001)
Protocol number	UCDCRC/20/01
EudraCT number	2020-001265-36
Objectives	To compare the efficacy of SOC plus hydroxychloroquine monotherapy or SOC plus a combination of hydroxychloroquine and azithromycin relative to standard of care (SOC) alone in the treatment of subjects with non-critical, SARS-CoV-2 PCR-positive infection not requiring immediate resuscitation or ventilation but who have evidence of clinical decline.
Study Design	Multi-centre, randomised, open label trial.<br><br>Subjects will be randomised 1:1:1 using a central register, with the use of permuted blocks of random sizes. To ensure concealment, the block sizes will not be disclosed.<br><br>All randomised participants are to be followed up until death, discharge from hospital or 60 days post-randomisation (whichever is sooner).
Subject Population	Non-critical, SARS-CoV-2 PCR-positive subjects not requiring immediate resuscitation or ventilation but who have evidence of progressive clinical decline<br><br>Inclusion Criteria<br>• Documented Covid-19 positive<br>• Aged ≥18 years male or female<br>• Evidence of clinical decline:<br>○ Elevated and/or rising (increases over 24 hours) inflammatory markers (at least two of CRP, d-dimer, LDH and/or ferritin above the upper limit of normal)<br>○ Presence of or progression of pulmonary infiltrates on CXR (as decided by the treating physician)<br>○ New hypoxia requiring ≥2l/min/28% FiO2 to maintain oxygen saturations ≥94% (or 88-92% in patients with chronic hypercapnic respiratory failure)<br><br>Exclusion Criteria<br>• Pregnant or breastfeeding woman<br>• Known hypersensitivity to chloroquine or hydroxy chloroquine or any excipients

	• Known hypersensitivity to azithromycin, erythromycin or any of the macrolide or ketolide antibiotics or any of the excipients<br>• Known deficit in G6PD<br>• Known retinopathy<br>• Patient with history of cardiac arrythmia related to QT prolongation<br>• Suspected acute cardiogenic pulmonary oedema at the time of enrolment<br>• QTc >500ms on two consecutive ECG measurements at screening<br>• Hypokalaemia (<3.0mmol/L) or hyperkalaemia (>6.1mmol/L) at screening<br>• Hypocalcaemia (<2.1mmol/L) or hypercalcaemia (>2.6mmol/L) (corrected for albumin) at screening<br>• Subjects receiving medications with a significant QT prolongation potential (if these treatments cannot be discontinued)<br><br>Participants enrolled into this study may be co-enrolled into other studies and clinical trials where they meet the specific eligibility criteria.<br><br><u>Sample size calculation</u><br>Based on W. Guan et al, they observed that 6.1% of patients receiving standard care progress to an intensive care unit (ICU), use of mechanical ventilation, or death. Assuming a median time to the primary endpoint of 6 days (and 6.1% progression) in the standard of care arm, we would need to enrol approximately 351 (117 per group) participants to provide the study with 80% power to detect an increase in the median time to progression of at least 3 days in either treatment arms, with 2.5% type I error (to account for the two primary comparisons) for a two sided log-rank test.
Study Treatment	Patients will be randomised 1:1:1 with the use of permuted blocks of random sizes to:<br>1. Standard of care<br>2. Standard of care plus hydroxychloroquine (400mg BID on day 1 then 200mg twice daily from day 2 to 10)<br>3. Standard of care plus hydroxychloroquine (400mg BID on day 1 then 200mg twice daily from days 2 to 10) and azithromycin (500mg day 1 and 250mg daily from days 2 to 5)
Study Assessments	• Clinical and medical history<br>• Assessments of safety and records of adverse events<br>• Physical exam<br>• Vital signs<br>• ECG<br>• Chest X-ray<br>• Laboratory assessments including bloods, pregnancy test,

	COVID-19 test<br>• Sample storage
Endpoints	Composite primary endpoint is time to progression to intubation, non-invasive ventilation, use of immunomodulatory therapy for COVID19 infection or death.<br><br>Immunomodulatory therapy refers to use of high dose corticosteroids (methylprednisolone) or new initiation of any humanised monoclonal antibody or convalescent serum.<br><br>Secondary endpoints include:<br>• 14, 28 and 60 day all-cause mortality<br>• time to clearance of SARS-CoV-2 from nasopharyngeal area (PCR)<br>• change in inflammatory markers
Data Analysis	Characteristics of study participants will be summarized by treatment arm using mean (standard deviation), median (interquartile range) or count (percentage) as appropriate.<br><br>Comparisons will be made between all participants randomised to the different treatment arms, irrespective of whether they received their allocated treatment ("intention-to-treat" analyses).<br><br>Two comparisons of equal importance will be tested in the trial: SOC plus hydroxychloroquine monotherapy versus SOC and SOC plus a combination of hydroxychloroquine and azithromycin vs SOC. The composite primary-endpoint of time to progression to intubation and invasive mechanical ventilation, death will be evaluated /estimated using the Kaplan–Meier method and compared between the treatment arms using the log-rank test. Kaplan-Meier estimates will be reported for each of the arms, with 95% confidence intervals. To quantify treatment effects in the primary analysis, unadjusted hazard ratios (with 95% confidence intervals) will be provided, based on a Cox proportional hazards regression. Effects of subgroups (age, gender and baseline fraction of inspired oxygen (FiO_2)) will be evaluated on the treatment effects through use of interaction terms. Further cox models will be constructed to adjust for any additional prognostic factors that are imbalanced at baseline. Categorical data on treatment safety (including adverse events) will be compared between treatment arms using the chi-square tests.<br><br>Time to event secondary endpoint will be analysed using a similar approach as the primary endpoint. For the longitudinal analysis of the exploratory secondary endpoints of inflammation markers, linear mixed models will be used, with random slope and subject intercepts and with time and treatment and their interaction, as well as the

	baseline values, as fixed effects. Inflammation response markers will be log-transformed, if appropriate, before longitudinal analysis. A post-hoc procedure to account for multiplicity in tests of secondary endpoints, with a Bonferroni-adjusted significance level will be applied to any claim for benefit in the secondary end points.

Schedule of Visits

Study Visit	Screening	Baseline Day 1	Day 2	Day 3	Day 4	Day 5	Day 10 (± 1)	Day 14 (± 2)	Day 21 (± 3)	Day 28 (± 3)	Day 60 (± 7)
**Administrative procedures**											
Informed consent	X										
Randomisation		X									
Dispense study medication		X									
**Clinical/Medical assessments**											
Demographic, clinical and medical history	X										
Medication review			X	X		X	X				
Assessment of Safety[9]		X	X	X		X	X	X	X	X	X
Full physical examination	X										
Directed physical exam		X	X	X		X	X	X	X	X	X
Vital signs	X	X	X	X		X	X	X	X	X	X
ECG	X	X[8]	X[10]	X	X	X	X				
Chest X-ray	X		X			X	X	X[1]		X[1]	X[1]
**Laboratory Assessments**											
Swab for COVID-19 PCR	X[2]					X	X	X			
FBC and differential		X	X	X		X	X		X		

Study Visit	Screening	Baseline Day 1	Day 2	Day 3	Day 4	Day 5	Day 10 (± 1)	Day 14 (± 2)	Day 21 (± 3)	Day 28 (± 3)	Day 60 (± 7)
Chemistry panel (Liver, renal and bone profile and CK)[3]		X	X	X		X	X		X		
Ferritin[4]	X		X	X		X	X		X		
Troponin	X		X	X		X	X		X		
D-dimer	X		X	X		X	X		X		
CRP	X		X	X		X	X		X		
LDH	X		X	X		X	X		X		
Arterial blood gas	X		X			X[5]	X[5]				
Pregnancy test (serum or urine βHCG)[6]	X					X					
**Sample Storage**											
Serum/plasma samples		X				X	X			X	X
Urine / stool samples		X					X			X	X
Respiratory samples		X		X		X	X	X[7]		X[7]	

1. If residual changes observed on previous CXR
2. If a positive result is not available from the previous 48 hours
3. Bilirubin, alkaline phosphatase, alanine aminotransferase, gamma glutamyl transferase, albumin; urea, creatinine; corrected calcium and phosphate and creatinine kinase
4. Serum iron, transferrin, total iron biding capacity, ferritin
5. If hypoxic (pO2 sats<95% on room air or in need of supplemental oxygen) and clinically indicated by the treating physician
6. For women of child-bearing potential or if pregnancy is suspected. If a urine pregnancy test is positive, it must be confirmed with a serum pregnancy test
7. If still an inpatient in hospital
8. If baseline visit is not conducted the same day as the screening visit
9. Specific assessments of new neuropsychiatric AEs will be made at baseline, day 5 and day 10
10. ECG to be performed 2-3 hours after dosing of IMP

Table of Contents

.... 1

PROTOCOL SIGNATURE PAGE 2

DOCUMENT HISTORY 3

ABBREVIATIONS 4

PROTOCOL SYNOPSIS 5

SCHEDULE OF VISITS 9

INTRODUCTION 13

BACKGROUND 13
PROPOSED TRIAL 13
INTERVENTIONS 13
POTENTIAL RISKS 15
POTENTIAL BENEFITS 16

TRIAL DESIGN 16

STUDY TREATMENTS 17

HYDROXYCHLOROQUINE 17
AZITHROMYCIN 20
SUPPLY, PACKAGING AND STORAGE 22

STUDY PROCEDURES 22

INFORMED CONSENT: 22
INCLUSION/EXCLUSION CRITERIA: 23
MEDICAL/CLINICAL HISTORY 24
CLINICAL ASSESSMENTS/PROCEDURES 25
ASSIGNMENT OF STUDY NUMBER 26
RANDOMISATION 26
STUDY OUTCOMES: 26
DURATION OF FOLLOW UP 27
SUBJECT WITHDRAWAL CRITERIA AND PROCEDURES FOR WITHDRAWAL 27

STUDY VISITS 28

SCREENING VISIT 28
BASELINE VISIT – RANDOMISATION VISIT 28
SUBSEQUENT VISITS 28
EARLY DISCONTINUATION VISIT 29

BIOLOGICAL SAMPLES 29

ANALYSIS 30

SAMPLE SIZE 30

SAFETY REPORTING REQUIREMENTS/DATA AND SAFETY MONITORING PLAN 31

DATA MANAGEMENT 34

DATA AND SAFETY MONITORING BOARD 36

REGULATORY APPROVAL 36

QUALITY CONTROL & QUALITY ASSURANCE PROCEDURES 37

FINANCING & INSURANCE/INDEMNITY 38

BIBLIOGRAPHY 39

APPENDIX A – HYDROXYCHLOROQUINE SUMMARY OF PRODUCT CHARACTERISTICS 40

APPENDIX B – AZITHROMYCIN SUMMARY OF PRODUCTS CHARACTERISTICS 51

APPENDIX C – STORAGE OF SAMPLES **63**
APPENDIX D - ADVERSE EVENTS AND SERIOUS ADVERSE EVENTS **68**
APPENDIX E – LABORATORY REFERENCE RANGES **71**
APPENDIX F – SAMPLE DRUG LABELS **75**
APPENDIX G – DECLARATION OF HELSINKI **76**

INTRODUCTION

Background

On 31 December 2019, China reported cases of pneumonia from a previously unknown coronavirus, 2019-nCoV, also called SARS-CoV-2. The disease it causes has been called COVID-19. The World Health Organization (WHO) designated COVID-19 a "public health emergency of international concern" on 30 January 2020 and declared it a pandemic on 11 March 2020.

Coronaviruses are single-stranded RNA viruses that encode for four enzymes essential to the viral life cycle. They enter mammalian cells through an interaction of a viral spike glycoprotein and a receptor angiotensin 1 converting enzyme 2 (ACE2) for SARS-CoV-1 and SARS-CoV-2 and dipeptidyl peptidase 4 for MERS. Attempts to develop effective treatments against two other coronavirus diseases, SARS and MERS, have so far been unsuccessful. The COVID-19 outbreak is intensifying and spreading globally, to date over 500 trials have been registered on the WHO's International Clinical Trials Registry Platform, in an attempt to affect morbidity and mortality and help us understand the disease.

Proposed Trial

The aim of our study is to compare the efficacy of standard of care versus standard of care with hydroxychloroquine monotherapy or standard of care with a combination of hydroxychloroquine and azithromycin in the treatment of subjects with non-critical, SARS-CoV-2 PCR-positive infection (not requiring immediate resuscitation or ventilation) but who have evidence of progressive clinical decline as defined as at least two of the following:

- Elevated and/or rising (increases over 24 hours) inflammatory markers (at least two of CRP, d-dimer, LDH and/or ferritin above the upper limit of normal)
- Presence of or progression to pulmonary infiltrates on CXR
- Hypoxia (requirement for supplemental oxygen and an increase in oxygen requirements over at least 24 hours to maintain oxygen saturations above 95%)

Composite primary endpoint is time to progression to intubation, non-invasive ventilation, use of immunomodulatory therapy for COVID19 infection or death. Secondary endpoints include 14, 28 and 60 day all-cause mortality, time to clearance of SARS-CoV-2 from nasopharyngeal area (PCR), time to extubation, change from baseline in inflammatory markers, length of stay in ICU (where relevant) and total hospital length of stay.

Immunomodulatory therapy refers to use of high dose corticosteroids (methylprednisolone) or new initiation of any humanised monoclonal antibody or convalescent serum.

Interventions

Initially developed as an antimalarial drug but now more commonly used for treatment of rheumatoid arthritis, hydroxychloroquine, a less-toxic derivative of chloroquine, has attracted considerable interest as a potential therapy for COVID-19 infection. As it has immunomodulatory effects [1] [2-5], hydroxychloroquine is an appealing option mainly because it is an off-the-shelf drug, is available in low-cost generic versions, and has already been tested and approved for use in common conditions

such as malaria and arthritis. Furthermore, hydroxychloroquine's clinical safety profile is better than that of chloroquine, allowing a higher daily dose [6] and has fewer concerns about drug-drug interactions [7].

Hydroxychloroquine is thought to block infections by SARS-CoV-2 by inhibiting glycosylation of viral ACE-2 or inhibition of quinone reductase 2, reducing synthesis of viral sialic acid. In the previous SARS outbreak, hydroxychloroquine was reported to have anti-SARS-CoV activity in vitro [8]. This suggests that hydroxychloroquine may be a potential pharmacological agent for the treatment of COVID-19 infection.

Clinical trials of hydroxychloroquine treatment for COVID-19 pneumonia are underway in China (NCT04261517 and NCT04307693). The first study (NCT04261517) has shown positive preliminary outcomes although findings were not conclusive because of the small sample size.

Chloroquine and hydroxychloroquine may inhibit coronavirus replication through a series of steps. Firstly, the drugs can change the pH at the surface of the cell membrane and thus, inhibit the fusion of the virus to the cell membrane. They can also inhibit nucleic acid replication, glycosylation of viral proteins, virus assembly, new virus particle transport, virus release and other processes to achieve its antiviral effects [9]. Studies revealed that they also have potential broad-spectrum antiviral activity by increasing endosomal pH required for virus/cell fusion, as well as interfering with the glycosylation of cellular receptors of SARS-CoV [10, 11]. The anti-viral and anti-inflammatory activities of chloroquine may account for its potent efficacy in treating patients with COVID-19 pneumonia.

In a recent *in vitro* study, chloroquine was found to inhibit the growth of SARS-CoV-2 in vitro [12]. This finding has been supported by clinical studies conducted in approximately one-hundred SARS-CoV-2 infected patients (Huang J. Efficacy of Chloroquine and Lopinavir/ Ritonavir in mild/general novel coronavirus (CoVID-19) infections: a prospective, open-label, multicenter randomized controlled clinical study. 13 Feb 2020. http://www.chictr.org.cn/showproj.aspx?proj=49263).

Results from the *in vitro* study showed that both chloroquine and hydroxychloroquine have good antiviral activity and were found to decrease the viral replication in a concentration-dependent manner [7]. In this study, hydroxychloroquine exhibited better in vitro anti-SARS-CoV-2 activity than chloroquine. This was demonstrated by the EC_{50} values for hydroxychloroquine always being smaller than the EC_{50} values for chloroquine, indicating that hydroxychloroquine has a more potent antiviral activity. On the basis of hydroxychloroquine's superior antiviral and prophylactic activity, as well as its more tolerable safety profile in comparison to chloroquine, the authors believe that hydroxychloroquine may be a promising drug for the treatment of SARS-CoV-2 infection [13].

Recently Gautret et al., in their evaluation of hydroxychloroquine, found that the drug was "efficient" in clearing upper airways of the virus in three to six days in most patients (Gautret et al. (2020) Hydroxychloroquine and azithromycin as a treatment of COVID-19: results of an open-label non-randomized clinical trial. International Journal of Antimicrobial Agents – In Press 17 March 2020 – DOI: 10.1016/j.ijantimicag.2020.105949). The authors also recommended "that COVID-19 patients be treated with hydroxychloroquine and azithromycin to cure their infection and to limit the

transmission of the virus to other people in order to curb the spread of COVID-19 in the world." In this study, patients with confirmed COVID-19 infections were included in a single arm protocol to receive 600mg of hydroxychloroquine daily and their viral load in nasopharyngeal swabs was tested daily in a hospital setting. Depending on their clinical presentation, azithromycin was added to the treatment. Untreated patients from another centre and cases refusing the protocol were included as negative controls. Presence and absence of virus at Day 6 post inclusion was considered the end point.

Results showed that, six patients were asymptomatic, 22 had upper respiratory tract infection symptoms and eight had lower respiratory tract infection symptoms. Twenty cases were treated in this study and showed a significant reduction of the viral carriage at D6-post inclusion compared to controls, and much lower average carrying duration than reported of untreated patients in the literature. Azithromycin added to hydroxychloroquine was significantly more efficient for virus elimination. Despite the small sample size the authors concluded that hydroxychloroquine treatment is significantly associated with viral load reduction/disappearance in COVID-19 patients and its effect is reinforced by azithromycin.

A recent paper reported an inhibitor effect of remdesivir (an antiviral drug) and chloroquine on the growth of SARS-CoV-2 *in vitro,* [12] and an early clinical trial conducted in COVID-19 Chinese patients, showed that chloroquine had a significant effect, both in terms of clinical outcome and viral clearance, when comparing to controls groups [14]. Chinese experts recommend that patients diagnosed as mild, moderate and severe cases of COVID-19 pneumonia and without contraindications to chloroquine, be treated with 500mg chloroquine twice a day for ten days.

Potential Risks

The primary risks of this study are related to the potential adverse effects associated to the study drugs and study procedures.

The more common adverse effects associated with hydroxychloroquine and azithromycin are described in the protocol within the Section 'Study Treatments' and a full list of undesirable effects is provided within Appendix A and B (Section 4.8 of Summary of Product Characteristics).

The risks associated with the study procedures include:

Blood sampling: This procedure may be uncomfortable but rarely results in any significant problems. Side-effects that have been noted with drawing blood include feeling light-headed or faint, fainting, formation of a blood clot, bruising and/or infection at the site of the needlestick.

Chest-X-ray (CXR): The radiation X-rays produces can harm living tissues. This risk is relatively small, but it increases with cumulative exposure. As part of everyday living, everyone is exposed to naturally occurring background radiation and receives a dose of about 2 to 3 millisieverts (mSv) per year. This study will involve exposure to a very small dose of radiation. The total effective dose from this study is of 0.4-0.7 mSv. At this level, no harmful effects of radiation have been demonstrated, and the risk is very low.

Electrocardiogram (ECG): ECGs are safe, non-invasive, painless tests and have no major risks. The electrodes that connect the sensors to the chest do not send out electric shocks. Some people may

experience a skin rash where electrodes were placed. If any paste or gel was used to attach the electrodes, the subject may have an allergic reaction to it. The irritation usually goes away once the patches are removed without requiring treatment.

There are no identifiable psychological, sociological, economical or legal risks to the participants.

Potential Benefits

So far, an effective treatment against COVID-19 is yet to be found. Preliminary studies have suggested that hydroxychloroquine and azithromycin are potential pharmacological agents for the treatment of COVID-19 infection. The information gained from this study may be of benefit in the treatment of other people infected with COVID-19 and in the understanding of markers of disease progression.

TRIAL DESIGN

This trial is a multi-centre, randomised, open label trial that will recruit inpatients with confirmed COVID-19 infection who are non-critical (not requiring immediate resuscitation or ventilation) but who have evidence of progressive clinical decline as defined as at least two of the following:

1. Elevated and/or rising (increases over 24 hours) inflammatory markers (at least two of CRP, d-dimer, LDH and/or ferritin above the upper limit of normal)
2. Presence of or progression of pulmonary infiltrates on CXR (as decided by the treating physician)
3. New hypoxia requiring ≥2l/min/>28% FiO2 to maintain oxygen saturations ≥94% (or 88-92% in patients with chronic hypercapnic respiratory failure) .

Patients will be randomised 1:1:1, with the use of permuted blocks of random sizes to:
1. Standard of care
2. Standard of care plus hydroxychloroquine (400mg BID on day 1 then 200mg twice daily from days 2 to 10)
3. Standard of care plus hydroxychloroquine (400mg BID on day 1 then 200mg twice daily from days 2 to 10) and azithromycin (500mg day 1 and 250mg daily from days 2 to 5)

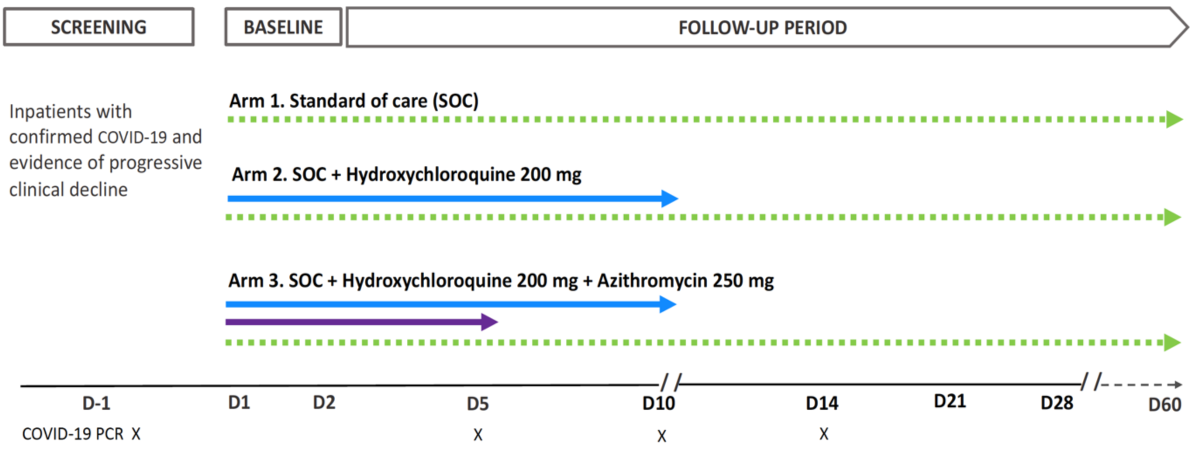

Primary outcome:

Composite primary endpoint is time to progression to intubation, non-invasive ventilation, use of immunomodulatory therapy for COVID19 infection or death.

<u>Secondary outcomes include:</u>

1. All-cause mortality at 14, 28 and 60 days
2. Time to clearance of COVID19 from nasopharyngeal pathway (PCR)
3. Change in inflammatory markers at day 7, 14 and 28
4. Change in supplemental oxygen requirements at day 7, 14 and 28
5. Length of stay in ICU
6. Time from intubation to extubation
7. Progression to moderate to severe respiratory failure as defined by $PaO_2/FiO_2 \leq 200mmHg$ and PEEP $\geq 5cmH_2O$
8. Time to discharge from hospital
9. Change/normalisation of A-a gradient
10. Resolution of radiological infiltrates

STUDY TREATMENTS

**Study Drug**	**Hydroxychloroquine**	**Hydroxychloroquine**	**Azithromycin**
**Dose Strength**	400mg	200mg	250mg
**Dose Regimen**	BID	BID	2 tablets once daily on day 1<br>1 tablet daily days 2-5
**Route of Administration**	Oral	Oral	Oral
**Duration of Treatment**	Day 1	Day 2-10	Day 1-5

Refer to ***Appendix A and B - Summary of Product Characteristics (SmPC)*** for full information, list of side effects and drug-drug interactions. University of Liverpool have developed an online database for checking drug-drug interactions with the experimental COVID-19 specific medicinal products; available online at www.covid19-druginteractions.org

Hydroxychloroquine

Administration instructions

- Hydroxychloroquine tablets are for oral administration.
- Each dose should be taken with a meal or glass of milk.

Contraindications

- known hypersensitivity to 4-aminoquinoline compounds
- pre-existing maculopathy of the eye
- pregnancy
- below 6 years of age (200mg tablets not adapted for weight <35kg)

Monitoring:

- ECG: hydroxychloroquine has been shown to prolong the QTc interval in some patients. Use with caution in patients receiving concomitant drugs known to prolong the QT interval or where a drug interaction may increase hydroxychloroquine exposure *(refer to Appendix A – Section 4.5 of the Summary of Product Characteristics)*
- Full Blood Count: Myelosuppression may occur rarely; monitor if pre-existing myelosuppression or if receiving other myelosuppressive agents concomitantly.
- Blood glucose: hydroxychloroquine may cause hypoglycaemia.

Special warnings and precautions for use

- Renal/hepatic function: hydroxychloroquine should be use with caution in patients with renal/hepatic impairment and those taking medicines known to affect those organs.
- Caution advised in patient with G6PD deficiency, may be risk of haemolysis. If status unknown, do not delay initiation of treatment in the context of moderate or severe COVID-19.
- Patients with severe gastrointestinal, neurological or blood disorders.
- Patients with porphyria cutanea tarda which can be exacerbated by hydroxychloroquine, and in patients with psoriasis since it appears to increase the risk of skin reactions.
- Patients with a sensitivity to quinine
- Due to low risk with recommended dose and duration of treatment, ophthalmological examination not required in context of COVID-19 infection.

Interactions with other medicinal products

- Digoxin: Hydroxychloroquine sulphate has been reported to increase plasma digoxin levels. Serum digoxin levels should be closely monitored in patients receiving concomitant treatment.
- Ciclosporin: An increased plasma ciclosporin level was reported when ciclosporin and hydroxychloroquine were co-administered. Ciclosporin levels should be monitored in patients receiving concomitant treatment.
- Halofantrine prolongs the QT interval and should not be administered with other drugs that have the potential to induce cardiac arrhythmias, including hydroxychloroquine.
- Amiodarone: there may be an increased risk of inducing ventricular arrhythmias if hydroxychloroquine is used concomitantly with other arrhythmogenic drugs, such as amiodarone and moxifloxacin.
- Antiepileptic drugs: The activity of antiepileptic drugs might be impaired if co-administered with hydroxychloroquine. Also, administration of hydroxychloroquine with antimalarials known to lower the convulsion threshold (e.g. mefloquine) may increase the risk of convulsions.
- Possible potentiation of neuromuscular blockade with aminoglycoside antibiotics.
- Antacids: antacids may reduce absorption of hydroxychloroquine, so it is advised that a four-hour interval be observed between hydroxychloroquine and antacid dosing.

- Insulin or antidiabetic drugs: as hydroxychloroquine may enhance the effects of a hypoglycaemic treatment, a decrease in doses of insulin or antidiabetic drugs may be required.
- There is a theoretical risk of inhibition of intra-cellular α-galactosidase activity when hydroxychloroquine is co-administered with agalsidase.
- Thyroid medication: hydroxychloroquine may increase thyroid stimulation hormone levels with concomitant levothyroxine.
- Avoid concomitant use of HCQ with drugs known to induce retinal toxicity.

Further information on interactions with other medicinal products is provided on Appendix A (Section 4.5 of the Summary of Product Characteristics).

More common undesirable effects

Metabolism and nutrition disorders
Common: Anorexia

Psychiatric disorders
Common: Affect lability

Nervous system disorders
Common: Headache

Eye disorders
Common: Blurring of vision due to a disturbance of accommodation which is dose dependent and reversible

Gastrointestinal disorders
Very common: Abdominal pain, nausea
Common: Diarrhoea, vomiting
These symptoms usually resolve immediately on reducing the dose or on stopping the treatment.

Skin and subcutaneous tissue disorders
Common: Skin rash, pruritus
These usually resolve readily on stopping treatment.

Full list of undesirable effects is provided within Appendix A (Section 4.8 of Summary of Product Characteristics).

Overdose
Overdosage with the 4-aminoquinolines is dangerous particularly in infants, as little as 1-2g having proved fatal.

The symptoms of overdosage may include headache, visual disturbances, cardiovascular collapse, convulsions, hypokalaemia, rhythm and conduction disorders, including QT prolongation, torsade de pointes, ventricular tachycardia and ventricular fibrillation, followed by sudden and potentially fatal respiratory and cardiac arrest. Immediate medical attention is required, as these effects may appear

shortly after the overdose. The stomach should be immediately evacuated, either by emesis or by gastric lavage.

Activated charcoal in a dose at least five times that of the overdosage may inhibit further absorption if introduced into the stomach by tube, following lavage, and within 30 minutes of ingestion of the overdose.

Consideration should be given to administration of parenteral diazepam in cases of overdosage; it has been shown to be beneficial in reversing chloroquine cardiotoxicity.

Respiratory support and shock management should be instituted as necessary.

Azithromycin

Administration instructions

- Azithromycin tablets are for oral administration as a single daily dose.

Contraindications

- Hypersensitivity to azithromycin, erythromycin or any of the macrolide or ketolide antibiotics or any of the excipients listed on the SmPC *(see Appendix B*)
- Not suitable for children under 45 kg body weight

Monitoring

- ECG: prolonged cardiac repolarisation and QT interval, imparting risk of developing cardiac arrhythmia and torsades de pointes, have been seen in treatment with azithromycin and other macrolides *(refer to Appendix B – Section 4.4 of the Summary of Product Characteristics)*

Special warnings and precautions for use

- Elderly patients: the same dose as in adults is used in the elderly. Since elderly patients can be patients with ongoing proarrhythmic conditions a particular caution is recommended due to the risk of developing cardiac arrythmia and torsades de pointes.

- Renal impairment: caution should be exercised when azithromycin is administered to patients with GFR<10 ml/min, a 33% increase in systemic exposure to azithromycin was observed. No dose adjustment is necessary in patients with mild to moderate renal impairment (GFR > 40 ml/min)

- Hepatic impairment: since azithromycin is metabolised in the liver and excreted in the bile, the drug should not be given to patients suffering from severe liver disease.

- Myasthenia gravis: exacerbations of the symptoms of myasthenia gravis and new onset of myasthenia syndrome have been reported in patients receiving azithromycin therapy.

- Superinfection: as with any antibiotic preparation, observation for signs of superinfection with non-susceptible organisms, including fungi is recommended. Clostridium difficile-associated diarrhoea (CDAD) have been reported with the use of nearly all antibacterial agents, including azithromycin. CDAD must be considered in all patients who present with diarrhoea following antibiotic use. Careful medical history is necessary since CDAD has been reported to occur over 2 months after the administration of antibacterial agents.

Interactions with other medicinal products

- Digoxin: azithromycin might increase digoxin plasma concentrations. Clinical monitoring and possible serum digoxin levels during treatment with azithromycin and after its discontinuation are necessary.
- Ciclosporin: azithromycin 500mg/day oral dose for 3 days with single 10mg/kg oral dose of ciclosporin showed to increase ciclosporin Cmax and AUC (0-5) by 24%. If co-administration is necessary, ciclosporin levels should be monitored and dose adjusted accordingly.
- Antacids: azithromycin should be taken at least 1 hour before or 2 hours after the antacid.
- Statins: post-marketing cases of rhabdomyolysis in patients receiving azithromycin with statins have been reported.
- Coumarin-type oral anticoagulants: potentiated anticoagulation subsequent to co-administration of azithromycin and coumarin-type oral anticoagulants has been reported. consideration should be given to the frequency of monitoring prothrombin time when azithromycin is used in patients receiving coumarin-type oral anticoagulants.

Further information on interactions with other medicinal products is provided on Appendix B (Section 4.5 of the Summary of Product Characteristics).

More common undesirable effects
Nervous system disorders
Common: Headache, dizziness, somnolence, dysgeusia, paraesthesia

Eye/ear disorders:
Common: visual impairment, deafness

Gastrointestinal disorders
Very common: diarrhoea, abdominal pain, nausea, flatulence
Common: vomiting, dyspepsia

Skin and subcutaneous tissue disorders:
Common: rash, pruritus

Musculoskeletal and connective tissue disorders:
Common: arthralgia

General disorders and administration site conditions:
Common: fatigue

Investigations
Common: eosinophil, basophils, monocytes and neutrophils count increased; lymphocyte count decreased; blood bicarbonate decreased

Full list of undesirable effects is provided within Appendix B (Section 4.8 of the Summary of Product Characteristics).

Overdose

Adverse events experienced in higher than recommended doses were similar to those seen at normal doses. In the event of overdosage, general symptomatic and supportive measures are indicated as required.

Supply, Packaging and Storage

Hydroxychloroquine 200mg tablets and Azithromycin 250mg tablets/capsules will be obtained by each site's hospital pharmacy department through the usual supply chain. Any available brand will be ordered to ensure continuity of supply. Commercial products will be labelled by a designated study pharmacist to ensure compliance with Annex 13 requirements.

IMP will be stored in a secure place in accordance with GCP requirements and below 30 degrees Celsius at all times.

Simplified accountability logs will be used to record individual transactions to patients and patient returns, as applicable.

If patients do not complete their full course of IMP, drug returns may be obtained where possible in line with local infection control recommendations. In order to preserve national stock of hydroxychloroquine and azithromycin, unused IMP which has not left the hospital may be re-purposed if appropriate to avoid drug wastage.

STUDY PROCEDURES

The Schedule of Visits summarises the trial procedures to be performed at each visit. Individual trial procedures are described in detail below. It may be necessary to perform these procedures at unscheduled time points if deemed clinically necessary by the investigator.

Furthermore, additional evaluations/testing may be deemed necessary by the investigator and/or the Sponsor for reasons related to subject safety.

Informed Consent:

It is the responsibility of the Investigator to ensure that Subjects meet the eligibility criteria for the study.

The investigator must obtain documented, freely-given consent from each potential subject or each subject's legally acceptable representative prior to any protocol-specific procedures being conducted on that subject and after the subject has read the trial Patient Information Statement (PIS).

An oral explanation should be provided to the subject in addition to the written information and the investigator is responsible for ensuring that the subject understands all the aspects of the study prior to obtaining consent. The investigator may delegate part of this responsibility to the other site staff but should always be involved in the consent process.

Before informed consent may be obtained, the investigator, or a person designated by the investigator, should provide the subject or the subject's legally acceptable representative ample time and opportunity to inquire about details of the trial and to decide whether or not to participate in the trial. All questions about the trial should be answered to the satisfaction of the subject or the subject's legally acceptable representative.

Consent must be documented by the subject's dated signature or by the subject's legally acceptable representative's dated signature on a consent form along with the dated signature of the person conducting the consent discussion.

A copy of the PIS and a copy of the signed and dated consent form should be given to the subject or the subject's legally acceptable representative before participation in the trial. However, if this is not possible because of infection control risks associated with potentially contaminated consent forms, this can be deferred until a sufficient period of time has elapsed to ensure that this process is safe to perform. The consent process and most recent PIS and Consent Form version that has been approved by the ethics committee and HRCDC (where applicable) will be followed in any case.

If a subject is unable to read or if a legally acceptable representative is unable to read, an impartial witness should be present during the entire informed consent discussion. After the written informed consent form and any other written information to be provided to subjects, is read and explained to the subject or the subject's legally acceptable representative, and after the subject or the subject's legally acceptable representative has orally consented to the subject's participation in the trial and, if capable of doing so, has signed and personally dated the informed consent form, the witness should sign and personally date the consent form. By signing the consent form, the witness attests that the information in the consent form and any other written information was accurately explained to, and apparently understood by, the subject or the subject's legally acceptable representative, and that informed consent was freely given by the subject or the subject's legally acceptable representative.

In emergency situations, when prior consent of the subject is not possible, the consent of the subject's legally acceptable representative, if present, should be requested. When prior consent of the subject is not possible, and the subject's legally acceptable representative is not available, enrolment of the subject should require measures described in the protocol and/or elsewhere, with documented approval/favourable opinion by the IRB/IEC, to protect the rights, safety and well-being of the subject and to ensure compliance with applicable regulatory requirements. The subject or the subject's legally acceptable representative should be informed about the trial as soon as possible and consent to continue and other consent as appropriate should be requested.

Inclusion/Exclusion Criteria:

All Subjects must meet all of the inclusion criteria and none of the exclusion criteria to be eligible to participate in this study.

Inclusion Criteria

- Documented COVID-19 positive (positive PCR test on a respiratory sample for SARS-CoV2)
- Aged ≥18 years male or female
- Evidence of progressive clinical decline:
  - Rising inflammatory markers over a 24 hour period (increases in CRP, d-dimer, LDH and/or ferritin above the upper limit of normal)
  - Presence of or progression of pulmonary infiltrates on CXR (as decided by the treating physician)
  - New hypoxia requiring >2l/min/28% FiO_2 to maintain oxygen saturations ≥94% (or 88-92% in patients with chronic hypercapnic respiratory failure).

Exclusion Criteria

- Pregnant or breastfeeding woman
- Known hypersensitivity to chloroquine or hydroxychloroquine or any excipients (*full list of excipients provided on Appendix A and B - Section 6.1*)
- Known hypersensitivity to azithromycin, erythromycin or any of the macrolide or ketolide antibiotics or any of the excipients (*full list of excipients provided on Appendix A and B - Section 6.1*).
- Known deficit in G6PD (if status unknown, do not delay initiation of treatment in the context of moderate or severe COVID-19)
- Known retinopathy
- Patient with history of cardiac arrythmia related to QT prolongation
- Suspected acute cardiogenic pulmonary oedema at the time of enrolment
- QTc >500ms on two consecutive ECG measurements at screening
- Hypokalaemia (<3.0mmol/L) or hyperkalaemia (>6.1mmol/L) at screening
- Hypocalcaemia (<2.1mmol/L) or hypercalcaemia (>2.6mmol/L) (corrected for albumin) at screening
- Subjects receiving medications with a significant QT prolongation potential (if these treatments cannot be discontinued)

Participants enrolled into this study may be co-enrolled into other studies and clinical trials where they meet the specific eligibility criteria.

Medical/clinical history

Demographics:

Subjects' demographic data will be collected. The data required from all subjects include; sex, age, ethnic group, race, social living conditions, smoking (including vaping) and alcohol consumption.

COVID-19 History:

A detailed history will be obtained from the Subject on transmission source of COVID-19, symptoms experienced and date and time of COVID-19 diagnosis.

Medical History:

Medical history including date of onset of any symptoms, date of onset of fever (if known), date of first reported breathlessness and date of confirmed COVID-19 diagnosis to be recorded at screening visit in the source notes.

Concomitant medications:

Clinically significant drug-drug interactions may occur with the medicinal products used to treat COVID-19. A thorough medication history (including alternative and herbal medicines) should be obtained prior to initiation of treatment.

All medications including prescription medication, over the counter medications and herbal remedies to be recorded. Start dates, dose and frequency of each medication to be included where available.

Throughout the trial, all concomitant medications (including oxygen therapy) with start/ stop dates will be recorded.

Clinical Assessments/procedures

Physical Exam

A complete physical exam will be performed at screening. Information from physical exam must be documented in the source notes. Height and weight to be recorded. Self -reported height and weight can be used if unable to complete at screening visit.

A targeted symptom directed physical exam can be completed at subsequent visits.

Clinically significant findings must be reported as medical history or an adverse event.

Vital signs

Vital signs to be collected at each study timepoint. Vital signs recorded by hospital staff can be used for study if recorded date and time of vital sign has been recorded. Vital signs to include systolic and diastolic blood pressure, heart rate, respirations, pulse oximetry oxygen saturations (include fraction of inspired oxygen (FiO_2) and route of oxygen therapy administration if applicable) and temperature.

ECG:

A 12 lead ECG will be obtained at screening and again at baseline, if the baseline visit is not conducted on the same day as screening. Interpretation of the ECG tracing must be made by a qualified doctor. Clinically significant findings must be reported as medical history or an adverse event. Additional ECG will be performed daily from day 1 to day 5 and again at day 10. On day 2, the ECG should be performed 2-3 hours post dose of IMP.

Where the QTc on an ECG exceeds 500ms or increases >60ms above the QTc estimated at screening / baseline ECG reading, the ECG should be repeated immediately. If the QTc on the repeat ECG remains above 500ms or >60ms above the baseline value, the study medications should be immediately discontinued and not restarted. The subject will be offered to continue in the study follow-up.

Chest X-ray:

A chest x-ray will be obtained as outlined on admission to hospital as well as at day 2, day 5 and day 10. Chest X-rays on days 14, 28 and 60 should be undertaken if residual changes are observed on chest x-ray from previous visits. Clinically significant findings must be reported as medical history or an adverse event.

Pregnancy test:

Female subjects of childbearing potential must have a negative serum or urine pregnancy test (βHCG) to be eligible to participate in this study. If a urine pregnancy test is positive, it must be confirmed with a serum pregnancy test. A pregnancy test will be performed at the screening visit and at day 5 to exclude pregnancy.

Adverse events:

Adverse events will be recorded as per schedule of events. The Investigator is responsible for assessing adverse events and serious adverse events for causality and severity. Refer to Safety Reporting Requirements/Data and Safety Monitoring Plan.

Laboratory Assessments:

Laboratory assessments will be carried out at the trial site's local laboratory. The timing for these laboratory procedures/assessments are outlined in the Schedule of Visits.

The following laboratory samples will be collected as per schedule of assessments:

1. Blood
- Haematology
- Chemistry panel (renal, liver (including AST and ALT) and bone panel)
- Creatinine kinase
- CRP
- LDH
- Ferritin
- Troponin
- D-dimer
- Blood culture (if clinically indicated)
- Arterial blood gas (if available)
- Pregnancy testing (blood or urine)

2. Nasopharyngeal swab for SARS-CoV-19 PCR testing
3. Sample storage (*see appendix C*)

Assignment of Study Number

All eligible subjects will be assigned a unique study number at screening. The study number identifies the subject for all procedures occurring after enrolment.

Randomisation

Eligible patients will be randomised using a central register in the ratio 1:1:1, with the use of permuted blocks of random sizes. To ensure concealment, the block sizes will not disclosed. Randomisation will be performed through an interactive web-based electronic data capturing database.

Arm 1. Standard of care

Arm 2. Standard of care plus hydroxychloroquine (400mg twice daily on day one followed by 200mg twice daily for 10 days)

Arm 3. Standard of care plus hydroxychloroquine (400mg twice daily on day one followed by 200mg twice daily for 10 days) and azithromycin (500mg day 1 followed by 250mg daily days 2-5)

The randomisation number identifies the subject alongside the study screening number for all procedures occurring after randomisation. Once a randomisation number is assigned to a subject, it can never be reassigned to another subject.

Study Outcomes:

Study reported outcomes will be collected from laboratory results and Subjects source notes. The following outcomes will be recorded:

- Date of intubation for mechanical ventilation support
- Date of initiation of non-invasive positive-pressure ventilation
- Date of death

- Use of immunomodulatory therapy for COVID19 infection or death. (Immunomodulatory therapy refers to use of high dose corticosteroids (methylprednisolone) or new initiation of any humanised monoclonal antibody or convalescent serum.)
- All-cause mortality at 14, 28 and 60 days
- Time to clearance of COVID19 from nasopharyngeal pathway (PCR)
- Change in inflammatory markers at day 7, 14 and 28
- Change in supplemental oxygen requirements at day 7, 14 and 28
- Length of stay in ICU
- Time from intubation to extubation
- Progression to moderate to severe respiratory failure as defined by $PaO2/FiO2 \leq 200mmHg$ and PEEP $\geq 5cmH2O$
- Time to discharge from hospital
- Change/normalisation of A-a gradient
- Resolution of radiological infiltrates

Duration of Follow up

All randomised participants are to be followed up until death, discharge from hospital or 60 days post-randomisation (whichever is sooner). It is recognised that in the setting of this trial, there may be some variability in exactly how many days post-randomisation, information on disease status is collected. This is acceptable and will be taken account of in the analyses and interpretation of results, the principle being that some information about post-randomisation disease status is better than none.

Subject withdrawal criteria and procedures for withdrawal

Subjects may withdraw consent at any time for any reason. However, as the analysis of the primary endpoint will be intention to treat, we will encourage all study participants to continue in the study follow-up even if they decide to discontinue the Investigational Medicinal Product (IMP) or if the investigator or the Sponsor decides to discontinue them from the IMP. A subject must have IMP discontinued where they are observed to have a prolonged QTc >500ms on two consecutive ECGs

In addition, a subject may be withdrawn by the investigator or the Sponsor if enrolment into the study is considered inappropriate, the study is terminated, or for administrative and/or other safety reasons.

A subject must be discontinued from the study for any of the following reasons:

- The subject or legal representative withdraws consent
- A physician investigator feels it is in the best interest of the subject to discontinue
- The subject, if female, has a confirmed positive urine pregnancy test

Subjects who discontinue the study prior to the last scheduled treatment visit should have an Early Discontinuation visit conducted (see section *Early Discontinuation Visit*).

STUDY VISITS

Screening Visit

After providing explicit informed consent, subjects will be reviewed in their primary treating centre where they will undergo screening for eligibility. A screening CRF will be completed outlining:

Detailed clinical and medical history including history of onset of symptoms related to COVID-19 (date of onset of any symptoms, date of onset of fever and date of onset of breathlessness if known)

Full physical exam and vital signs

ECG

Chest X-ray

Blood assessments: CRP, LDH, ferritin, troponin, D-dimer, ECG, arterial blood gas

Pregnancy testing (blood or urine) in women of child-bearing age

Nasopharyngeal swab for SARS-CoV-19 PCR testing (if positive result not available within the period of this symptomatic episode)

Management of screen failures

Subjects failing to meet the eligibility requirements will continue in care as per standard guidelines

Baseline Visit – Randomisation visit

Subjects will undergo:

- Review of eligibility
- Physical directed examination and vital signs
- Assessment of neuropsychological AE
- Blood assessments: haematology, clinical biochemistry (renal, liver and bone profile), CK
- ECG (if baseline visit and screening visit are not performed on the same day)
- Samples for storage: serum, plasma, urine, stool and respiratory samples

Randomisation will be performed through an interactive web-based electronic data capturing database which will provide each subject with a randomisation code.

Subsequent visits

The following assessments will be conducted at each visit according to the schedule of visits:

- clinical and medical assessment, including assessment of safety (new neuropsychological AE at day 5 and 10), review of medications and side effects
- blood assessments: haematology, clinical biochemistry (renal, liver and bone profile), CRP, CK, LDH, ferritin, troponin, D-dimer, arterial blood gas (only where indicated after day 5 visit)
- Nasopharyngeal aspirates or respiratory specimens
- Physical directed examination and vital signs
- ECG
- Chest X-ray (only where clinically indicated after day 10 visit)
- Blood, urine and stool samples for storage
- Pregnancy testing (blood or urine) in women of child-bearing age (day 5)

Early discontinuation visit

If a subject discontinues from study medication or withdraws from participating in the trial all applicable activities should be performed at the time of discontinuation.

Subjects who cease study medication will, wherever possible, continue to be followed-up according to the protocol study plan. If a subject revokes consent or follow-up, if possible, all assessments scheduled to the final visit shall be completed.

Any adverse events which are present at the time of discontinuation/withdrawal should be followed in accordance with the safety requirements outlined in Section Recording and Reporting Adverse Events.

At a minimum, collect the following information when a subject discontinues:

- The reason the subject discontinued
- The date of the last dose of study medications from the trial
- The date of the last assessment and/or contact
- (Serious) Adverse Events
- Final Assessments: Every effort should be made to ensure that all procedures and evaluations scheduled for the final study visit are performed including samples for storage.

BIOLOGICAL SAMPLES

Blood, urine, stool and respiratory samples will be stored to enable future studies.

Blood samples for serum and plasma storage as well as urine and stool samples will be taken at baseline and day 10, 14, 28 and 60. Respiratory samples will be taken for storage at baseline, day 3, 5, 10, 14 and 28 (*see schedule of visits*).

These samples will be processed centrally at CEPHR by experienced laboratory staff according to a standardised protocol.

The methods for collection and processing laboratory samples prior to analysis, shipping or storage are detailed in the study Laboratory Manual. It is the responsibility for the investigator at each site to ensure that all site staff who will be handling, packaging, and/or shipping biological samples understand and comply with International Air Transport Association (IATA) regulations relating to the handling and shipping of hazardous goods and/or diagnostic specimens.

All stored samples will be identified by a study code, designation of study visit and date of collection and data linkage to the subject's medical record number (MRN) will be contained on a password-protected computer file located on a secure research computer network.

Samples will be stored at -80°C in a dedicated, monitored sample storage facility within CEPHR. This facility includes 24-hour real-time remote monitoring of freezers with alarms linked to a number of CEPHR and UCD staff to ensure integrity of samples for the duration of the study.

ANALYSIS

All analyses for reports, presentations and publications will be prepared by the coordinating centre at the UCD Centre for Experimental Pathogen Host Research and the UCD Clinical Research Centre.

Sample size

The hypotheses were that the SOC (standard of care) plus hydroxychloroquine or the SOC plus hydroxychloroquine + azithromycin arm would result in an increase in median time to progression to ICU/death/ventilation, as compared with the standard of care arm alone. Based on W. Guan et al, they observed that 6.1% of patients receiving standard care progress to an intensive care unit (ICU), use of mechanical ventilation, or death. Assuming a median time to the primary endpoint of 6 days (and 6.1% progression) in the standard of care arm, we would need to enrol approximately 351 patients to provide the study with 80% power to detect a smallest increase in the median time to progression in the experimental arms of at least 3 days (HR=0.67), with 2.5% type I error (to account for multiplicity based on two primary comparisons) for a two sided log-rank test.

Outcomes

The **primary objective** is to provide reliable estimates of the effect of study treatments on in-hospital death, use of immunomodulatory therapy for COVID-19 infection, intubation or progression to need for positive-pressure non-invasive ventilation (with subsidiary analyses of cause of death).

Immunomodulatory therapy refers to use of high dose corticosteroids (methylprednisolone) or new initiation of any humanised monoclonal antibody or convalescent serum.

The **secondary objectives** are to assess the effects of study treatments on duration of hospital stay and on the need for (and duration of) ventilation; and the need for renal replacement therapy.

Methods of analysis

Characteristics of study participants will be summarized by treatment arm using mean (standard deviation), median (interquartile range) or count (percentage) as appropriate.

Comparisons will be made between all participants randomised to the different treatment arms, irrespective of whether they received their allocated treatment ("intention-to-treat" analyses). Two comparisons of equal importance will be tested in the trial: SOC plus hydroxychloroquine monotherapy versus SOC and SOC plus a combination of hydroxychloroquine and azithromycin vs SOC. The composite primary-endpoint of time to progression to in-hospital death, use of immunomodulatory therapy for COVID-19 infection, intubation or progression to need for positive-pressure non-invasive ventilation will be evaluated /estimated using the Kaplan–Meier method and compared between treatment arms using the log-rank test. Kaplan-Meier estimates will be reported for each of the arms, with 95% confidence intervals. To quantify treatment effect in the primary analysis, unadjusted hazard ratios (with 95% confidence intervals) will be provided, based on a Cox proportional hazards regression. Effects of subgroups (age group, gender and baseline fraction of inspired oxygen (FiO_2)) will be evaluated on treatment effects through use of interaction terms. Further cox models will be constructed to adjust for any additional prognostic factors that are

imbalanced at baseline. Categorical data on treatment safety (including adverse events) will be compared between treatment arms using the chi-square tests.

Time to event secondary endpoint will be analysed using a similar approach as the primary endpoint. For the longitudinal analysis of the exploratory secondary endpoints of inflammation markers, linear mixed models will be used, with random slope and subject intercepts and with time and treatment and their interaction, as well as the baseline values, as fixed effects. Inflammation response markers will be log-transformed, if appropriate, before longitudinal analysis. A post-hoc procedure to account for multiplicity in tests of secondary endpoints, with a Bonferroni-adjusted significance level will applied to any claim for benefit in the secondary end points.

SAFETY REPORTING REQUIREMENTS/DATA AND SAFETY MONITORING PLAN

Timely, accurate and complete reporting and analysis of safety information from clinical studies are crucial for the protection of patients and are mandated by regulatory agencies worldwide.

Data from all subjects entering the study will be included in the analysis of safety. The number of adverse events (AEs) and serious adverse events (SAEs) will be tabulated by severity and treatment received. All such events will be recorded on the adverse event case report forms. *(See appendix D - Adverse Events and Serious Adverse Events definition and E – Laboratory Reference Ranges*).

Evaluation of AEs and SAEs

Seriousness, causality, severity and expectedness should be evaluated.

Assessment of seriousness

The investigator should make an assessment of seriousness as defined.

- AE results in death
- AE is life-threatening

Medical and scientific judgement should be exercised in deciding whether expedited reporting is appropriate in other situations, such as important medical events that may not be immediately threatening or result in death or hospitalisation, but may jeopardise the subject or may require intervention to prevent one of the outcomes listed in the SAEs definition.

Assessment of causality

The investigator's assessment of causality must be made for all AEs (serious and non-serious).

All adverse events judged by either the investigator or the sponsor as having a reasonable suspected causal relationship to an investigational medicinal product qualify as adverse reactions to the study drug.

The causality assessment given by the investigator should not be downgraded by the sponsor.

The investigator must make an assessment of whether the AE/SAE is likely to be related to treatment according to the following definitions:

- **Unrelated:** Where an event is not considered to be related to the study medication.

- **Possibly:** Although a relationship to the study medication cannot be completely ruled out, the nature of the event, the underlying disease, concomitant medication or temporal relationship make other explanations possible.
- **Probably:** The temporal relationship and absence of a more likely explanation suggest the event could be related to the study medication.

All AEs/SAEs judged as having a reasonable suspected causal relationship (e.g. possibly, probably) to the study medication will be considered as adverse reactions (Ars) or serious adverse reactions (SARs) – *see appendix D and E.* All AEs/SAEs judged as being related (e.g. possibly, probably) to an interaction between the study medication and another medication will also be considered to be ARs/SAR.

Alternative causes such as natural history of the underlying disease, concomitant therapy, other risk factors and the temporal relationship of the event to the treatment should be considered.

Assessment of severity

The investigator will make an assessment of severity for each AE/SAE and record this on the CRF according to one of the following categories:

- **Mild:** An event that is easily tolerated by the subject, causes minimal discomfort and does not interfere with everyday activities.
- **Moderate:** An event that is sufficiently uncomfortable to interfere with normal everyday activities.
- **Severe:** An event that prevents normal everyday activities.

Assessment of expectedness

The expectedness of an adverse reaction will be determined by the sponsor according to the reference document. For the study drugs involved in this study which have a marketing authorization (commercial agent), the expectedness of an adverse event will be determined by whether or not it is listed in the summary of product characteristics (SmPC) – section 4.8 undesirable effects.

For this study, the current versions of Hydroxychloroquine and Azithromycin SPCs will be used to assess the expectedness of the event in the study (*See Appendix A and B*)

Relation to Study IMP

The investigator (or designee) must determine the likelihood that the IMP caused the adverse event. The investigator must record the causal relationship in the patients' notes, as appropriate, and report such an assessment in accordance with serious adverse reporting requirements, if applicable.

An adverse event is considered associated with the use of the IMP/treatment if the attribution is definitely, probably or possibly related.

Risk related to the study procedures

- Blood sampling: this procedure is uncomfortable but rarely results in any significant problems. Side-effects that have been noted with drawing blood include feeling light-headed or faint, fainting, formation of a blood clot, bruising and/or infection at the site of the needlestick.

- Chest-X-ray: the chest-X-ray will involve exposure to a very small of radiation. As part of everyday living, everyone is exposed to naturally occurring background radiation and receives a dose of about 2 to 3 millisieverts (mSv) per year. The total effective dose from this study is of 0.4-0.7 mSv. At this level, no harmful effects of radiation have been demonstrated, and the risk is very low.
- Electrocardiogram (ECG): ECGs are safe, non-invasive, painless tests and have no major risks. The electrodes that connect the sensors to the chest do not send out electric shocks. Some people may experience a skin rash where electrodes were placed. If any paste of gel was used to attach the electrodes, the subject may have an allergic reaction to it. The irritation usually goes away once the patches are removed without requiring treatment.

Procedures for Recording and Reporting Adverse Events

The site clinical investigator is responsible for the detection and documentation of events meeting the definition of AE or SAE as provided in this protocol.

Only non-serious Adverse Events which are considered related to the IMP will be recorded routinely on the Adverse Event eCRF from the time of informed consent (or enrolment into the study) up to 90 days after the last dose of study drug has been received. All Adverse Events meeting "serious" criteria should be recorded on the Adverse Event eCRF as per Section below.

Procedures for Recording and Reporting Serious Adverse Events

AEs meeting the definition of SAEs must be reported using the SAE Report Form located in the Investigator Site File (ISF). All AEs meeting "serious" criteria occurring in each patient should be reported from the time of informed consent (or enrolment into the study) up to 90 days after the last dose of study drug has been received.

The Investigator will submit to the UCD Pharmacovigilance unit, using the SAE report forms provided, all Serious Adverse Events in the study regardless of whether causality with the administration of study drug is suspected by the investigator. The Investigator will transmit the SAE reports by email to the pharmacovigilance email immediately i.e. defined as within 24 hours of a member of the research team becoming aware of the event(s).

Any SAEs occurring in patients after 90 days of the last dose of study drug, which are deemed by the investigator to be causally related to study drug should be forwarded to UCD Pharmacovigilance Unit as outlined above.

All SAE reports to be emailed immediately to: sae.reporting@ucd.ie

UCD Pharmacovigilance will acknowledge receipt of the SAE via email to the reporter and/or Investigator.

All SAEs should be monitored until they are resolved or are clearly determined to be due to a patient's stable or chronic condition or intercurrent illness(es). Information not available at the time of the initial report e.g. pending lab results must be documented on a follow-up SAE report form. Follow-up information should be sought and submitted as it becomes available. The follow-up information should describe whether the event has resolved or persists, if and how it was treated and whether the patient continues on study or has been withdrawn from treatment.

If unsure whether an event meets seriousness criteria, please contact the Pharmacovigilance unit at the above email address who will deal with your query and advise on specific reporting requirements.

Sponsor Responsibilities:

The Sponsor, UCD, will perform appropriate adverse event reporting for the study according to the applicable regulatory guidelines. UCD will submit local SUSARs on an expedited basis to the local regulatory authority and other Competent Authorities involved, concerned Ethics Committees (EC) and all participating investigators. UCD sends annual Development Safety Update Reports (DSUR) to the concerned regulatory authority and Ethics Committee. Investigators will be informed of SUSARs.

Procedures for Documenting and Reporting Pregnancies

Where a female subject is enrolled and becomes pregnant during the course of the study must be reported to UCD Pharmacovigilance unit by the investigator within 24 hours of the study site becoming aware of the event, by email using the Pregnancy Report Form located in the Investigator Site File (ISF). Forms should be sent to the email address sae.reporting@ucd.ie.

The Pharmacovigilance unit will acknowledge receipt of this form by email. Any patient who becomes pregnant during the study must be promptly withdrawn from the study following withdrawal procedures outlined in protocol section *'Study Procedures – subject withdrawal criteria and procedures for withdrawal'*. The pregnancy will be monitored closely until the birth. Follow-up information regarding the outcome of the pregnancy and any postnatal sequelae in the infant will be required.

Exposure during Pregnancy or Breastfeeding (even if not associated with an adverse event) should be reported.

DATA MANAGEMENT

The site investigator will facilitate the permit of study-related monitoring, audit, research ethics committee approval review and regulatory inspection by the relevant national authority, providing direct access to source documents were necessary.

Data will be generated and managed according to the study specific data management plan set out in the study site file. Sources of data will be identified and pre-specified on a study-specific source data agreement relevant to the study site. The source data agreement will be verified by the site Investigator with a copy of the agreement to be filed within the Site File.

The study data will be collected using Case Report Forms (CRFs). A case report form (CRF) is designed to record the data required by the protocol and collected by the investigator in the patient's clinical source documents.

Source documents for this study will include hospital records, procedure reports and data collection forms. Any data to be entered directly on the Case Report Forms will be considered to be source data. Source documents will be stored in a secure location as per hospital/site policy.

Data collected will be coded. On all study-specific documents other than the signed consent, the subject will be referred to by the study subject identification number/code. Any reports that acts as a

source document, other than completed CRF, should be signed and dated by a listed site investigator for issues of medical significance (for example the review of laboratory reports).

Subject participation and subject progress should also be recorded in the subject medical records to ensure relevant healthcare providers have knowledge of the subject's participation in the study.

Clinical trial data will be entered by authorised site personnel into the electronic data capture system. The data should be verifiable against original records and source notes by the Monitor during monitoring visits. Data reported on the eCRF that are derived from source documents must be consistent with the source documents or the discrepancies must be explained.

CRF completion instructions will be provided and any questions about recording specific information on the CRF should be directed to the Monitor.

Data Quality Assurance

All data will be reviewed for completeness and logical consistency by the Data Management team. Data queries will be generated via the electronic data capture system to correct or clarify data or request missing information. The designated site staff will be required to respond to these queries in accordance with data entry and data query timelines for the study.

The investigator will be responsible for the review and sign off of data entered and corrected for their site.

Record Retention

An investigator site file will be provided by UCD for all required study documents. The investigator will maintain all study records according to ICH-GCP and applicable regulatory requirement(s). Essential documents will be retained until at least 15 years after the publication of the clinical study report. These documents should be retained for a longer period however if required by the applicable regulatory requirements or by an agreement with the sponsor.

The investigator/institution should agree to retain the trial-related essential documents as required by the applicable regulatory requirements and until the sponsor informs the investigator/institution these documents are no longer necessary.

Confidentiality of Subject Records

By signing the protocol, the investigator agrees that the sponsor, ethics committee or regulatory authorities may consult and/or copy study documents to verify information in the case record form. By signing the consent form the subject agrees to this process.

Subject confidentiality will be maintained at all times and no documents containing the subject's name or other identifying information will be collected by the sponsor on the study database. It may be necessary for the sponsor's representatives, the ethics committee and regulatory authority representatives to have direct access to the subject's medical records. If study documents need to be photocopied during the process of verifying case record form data, the subject will be identified by a unique code only; full names and other identifying information will be masked.

Confidentiality of Study Data

By signing this protocol, the investigator affirms to the sponsor that information provided to the investigator by the sponsor will be maintained in confidence and will be divulged only as necessary to the ethics committee and institution employees directly involved in the study. Both ethics committee members and employees must also understand the confidentiality requirements for any information divulged to them. The data generated by this study will be considered confidential by the investigator, except to the extent that it is included in a publication as agreed in the publication policy of this protocol.

DATA AND SAFETY MONITORING BOARD

An independent Data and Safety Monitoring Board (DSMB) will be established to perform ongoing safety surveillance and to perform interim analyses on the study data.

The DSMB will be an independent committee, composed of a minimum of three members; at least two will be clinicians not involved in the trial but with experience and expertise in clinical trials and / or biostatistics; at least one member will be a clinician with expertise in infectious diseases. Each member will state that they have no conflict of interest with the sponsor involved in the study or any other conflicting interest to declare. The DSMB will be supported by the study statistician.

The DSMB will meet on two occasions; once 15 subjects have completed day 14 of study and once again when 50% subjects have completed day 14 of the study to decide if the safety of the planned intervention(s) is acceptable. The DSMB will be provided with relevant updated analyses of safety by the study statistician. Any imbalances in prevalence of and/or severity of adverse events between arms will be addressed an recommendations made to the sponsor to either:

1. Discontinue the clinical trial immediately due to concerns around safety
2. Discontinue one of the study arms where there are concerns around safety
3. Continue with the clinical trial

There will be no pre-planned interim analysis of efficacy.

The DSMB will consider findings from any other relevant studies and review trial data on recruitment, safety, and adherence to trial strategies and efficacy in strict confidence. Where new findings are published that may impact on the relevance of continuing the study or raise questions as to safe conduct of the trial, the sponsor may request additional DSMB meetings to consider such evidence. If they observe any significant excess of serious adverse events in any treatment group, they may recommend premature termination of the trial on the basis of serious safety concerns. The group will consider the progress of the study and make recommendations to the sponsor where necessary.

REGULATORY APPROVAL

Regulatory approval from the Health Products Regulatory Authority (HPRA) will be obtained prior to conduct of the clinical trial in Ireland. This study will be conducted in accordance with the regulatory requirements, sponsor standard operating procedures (SOPs), ICH GCP and ethical principles laid out

in the Declaration of Helsinki (2013) and the National Statement on Ethical Conduct in Research involving Humans *(See Appendix F)*.

The investigator is responsible for obtaining ethics committee approval of the protocol in compliance with the local regulatory requirements prior entering any subject into the clinical trial. The approval must clearly identify the protocol and all documents approved by the ethics committee including version numbers of the protocol and informed consent. A copy of the approval should be sent to the sponsor of the study.

The investigator should also obtain approval for any amendments to the protocol or informed consent during the course of the study. The investigator must comply with all ethics committee reporting requirements for serious adverse events, annual updates and end of study reports and must agree to abide by any ethics committee conditions of approval. The Ethics Committee must review and approve the initial and any amended consent forms prior to use in the study. The subject or the legally acceptable representative should be informed in a timely manner of any new information that becomes available during the course of the study that may affect the subject's willingness to continue participation in the trial. The communication of this information may be provided and documented via a patient letter or revised consent form or addendum to the original consent form that captures the subject's dated signature or by the subject's legally acceptable representative's dated signature.

Specifics about a trial and the trial population will be added to the consent form template at the protocol level.

The informed consent will adhere to IRB/ERC requirements, applicable laws and regulations and Sponsor requirements.

QUALITY CONTROL & QUALITY ASSURANCE PROCEDURES

The sponsor will conduct regular monitoring visits throughout the course of the clinical trial, in accordance with ICH GCP. Monitor will conduct a minimum of two source data verification visits at each clinical site during the course of the study. These will occur shortly after the entry of the first subject(s) into the study and at the end of the study once all subject visits have been completed. The investigator will allow the monitor to visit the site and facilities where the study will take place in order to ensure compliance with the protocol requirements and ICH GCP. Training sessions may be organised for the investigators and/or instruction manuals may be given to the study team as required.

The Investigator will permit the Sponsor, authorised agents of the Sponsor (such as Study Monitors, auditors, etc.) and regulatory agency employees to enter and inspect any site where the drug or records pertaining to the drug are held, and to inspect and copy all records relating to an investigation, including research participant records.

To ensure the accuracy of data submitted, it is mandatory that representatives of the Sponsor and of the regulatory agencies have access to source documents (i.e. participant medical records or notes, charts, laboratory reports, etc.). Participant confidentiality will be protected at all times. The investigator will allow the monitor to:

- inspect the site, the facilities and the material used for the study,
- meet all members of his/her team involved in the study,
- consult all of the documents relevant to the study,
- have access to the electronic case report forms (i.e. access to an analogic phone line or his/her computer)
- check that the electronic case report forms have been filled out correctly,
- directly access source documents for comparison of data therein with the data in the electronic case report forms,
- verify that the study is carried out in compliance with the protocol and local regulatory requirements.

All data will be stored securely. Details of outcome measures and adverse events will be documented in hospital healthcare records, in individual research participant case report forms and in an encrypted electronic database.

The study investigators will adhere to hospital protocols pertaining to healthcare record use and storage. The investigators and authorised designees will ensure that the confidentiality of the participants' data is preserved.

This trial may be subject to internal or external auditing or inspections procedure to ensure adherence to GCP. Access to all trial-related documents will be given at that time.

A quality assurance audit may be conducted by the sponsor or its agent at any time during, or shortly after, the study. The investigator will permit an independent audit by an auditor mandated by Sponsor, after reasonable notice. The purpose of an audit is to confirm that the study is conducted as per protocol, GCP and applicable regulatory requirements, that the rights and well-being of the patients enrolled have been protected, and that the data relevant for the evaluation of the investigational medicinal product have been captured, processed and reported in compliance with the planned arrangements. The investigator will permit direct access to all study documents, drug accountability records, medical records and source data.

Regulatory authorities may perform an inspection of the study up to several years after its completion. If an inspection is announced the Sponsor will be informed immediately.

FINANCING & INSURANCE/INDEMNITY

The UNIVERSITY COLLEGE OF DUBLIN is the SPONSOR and it will ensure that every investigator is covered by a Public Liability ('negligent harm') insurance that applies for the clinical trial. All investigators are qualified and practicing physicians and are thus insured by the clinical indemnity scheme (CIS).

University College Dublin is funding this trial.

All relevant insurance documentation will be obtained prior to conduct of the trial.

Bibliography

1. Romanelli, F., K.M. Smith, and A.D. Hoven, *Chloroquine and hydroxychloroquine as inhibitors of human immunodeficiency virus (HIV-1) activity.* Curr Pharm Des, 2004. **10**(21): p. 2643-8.
2. Keyaerts, E., et al., *In vitro inhibition of severe acute respiratory syndrome coronavirus by chloroquine.* Biochem Biophys Res Commun, 2004. **323**(1): p. 264-8.
3. Vincent, M.J., et al., *Chloroquine is a potent inhibitor of SARS coronavirus infection and spread.* Virol J, 2005. **2**: p. 69.
4. Ooi, E.E., et al., *In vitro inhibition of human influenza A virus replication by chloroquine.* Virol J, 2006. **3**: p. 39.
5. Li, C., et al., *Chloroquine, a FDA-approved Drug, Prevents Zika Virus Infection and its Associated Congenital Microcephaly in Mice.* EBioMedicine, 2017. **24**: p. 189-194.
6. Marmor, M.F., et al., *Recommendations on Screening for Chloroquine and Hydroxychloroquine Retinopathy (2016 Revision).* Ophthalmology, 2016. **123**(6): p. 1386-94.
7. Yao, X., et al., *In Vitro Antiviral Activity and Projection of Optimized Dosing Design of Hydroxychloroquine for the Treatment of Severe Acute Respiratory Syndrome Coronavirus 2 (SARS-CoV-2).* Clin Infect Dis, 2020.
8. Biot, C., et al., *Design and synthesis of hydroxyferroquine derivatives with antimalarial and antiviral activities.* J Med Chem, 2006. **49**(9): p. 2845-9.
9. Fox, R.I., *Mechanism of action of hydroxychloroquine as an antirheumatic drug.* Semin Arthritis Rheum, 1993. **23**(2 Suppl 1): p. 82-91.
10. Savarino, A., et al., *Effects of chloroquine on viral infections: an old drug against today's diseases?* Lancet Infect Dis, 2003. **3**(11): p. 722-7.
11. Yan, Y., et al., *Anti-malaria drug chloroquine is highly effective in treating avian influenza A H5N1 virus infection in an animal model.* Cell Res, 2013. **23**(2): p. 300-2.
12. Wang, M., et al., *Remdesivir and chloroquine effectively inhibit the recently emerged novel coronavirus (2019-nCoV) in vitro.* Cell Res, 2020. **30**(3): p. 269-271.
13. Furst, D.E., *Pharmacokinetics of hydroxychloroquine and chloroquine during treatment of rheumatic diseases.* Lupus, 1996. **5 Suppl 1**: p. S11-5.
14. Gao, J., Z. Tian, and X. Yang, *Breakthrough: Chloroquine phosphate has shown apparent efficacy in treatment of COVID-19 associated pneumonia in clinical studies.* Biosci Trends, 2020. **14**(1): p. 72-73.
15. Sheahan, T.P., et al., *Comparative therapeutic efficacy of remdesivir and combination lopinavir, ritonavir, and interferon beta against MERS-CoV.* Nat Commun, 2020. **11**(1): p. 222.
16. Cao, B., et al., *A Trial of Lopinavir-Ritonavir in Adults Hospitalized with Severe Covid-19.* N Engl J Med, 2020.
17. Wang, Y., et al., *Comparative effectiveness of combined favipiravir and oseltamivir therapy versus oseltamivir monotherapy in critically ill patients with influenza virus infection.* J Infect Dis, 2019.

Appendix A – Hydroxychloroquine Summary of Product Characteristics

1 NAME OF MEDICINAL PRODUCT

Plaquenil 200mg Film-coated Tablets

2 QUALITATIVE AND QUANTITATIVE COMPOSITION

Each tablet contains 200 mg Hydroxychloroquine sulphate.

Excipients:
Lactose monohydrate 35.25 mg per tablet

For full list of excipients, see section 6.1.

3 PHARMACEUTICAL FORM

Film-coated tablet.
White, biconvex tablets with flat sides, marked HCQ on one side and 200 on the reverse.

4. CLINICAL PARTICULARS

4.1 Therapeutic indications

Adults
Plaquenil tablets are recommended for the treatment of rheumatoid arthritis, discoid and systemic lupus erythematosus, and dermatological conditions caused or aggravated by sunlight.

Paediatric Population
Treatment of juvenile idiopathic arthritis (in combination with other therapies), discoid and systemic lupus erythematosus.

4.2 Posology and method of administration

Plaquenil tablets are for oral administration. Each dose should be taken with a meal or glass of milk.

Hydroxychloroquine is cumulative in action and will require several weeks to exert its beneficial effects, whereas minor side effects may occur relatively early.

For rheumatic disease treatment should be discontinued if there is no improvement by 6 months.
In light-sensitive diseases treatment should only be given during periods of maximum exposure to light.

Adults (including the elderly)

The minimum effective dose should be employed. This dose should not exceed 6.5mg/kg/day (calculated from ideal body weight and not actual body weight) and will be either 200mg or 400mg per day. The 400mg tablet should not be used in adults with an ideal body weight of less than 62kg.

Paediatric Population

The minimum effective dose should be employed and should not exceed 6.5mg/kg/day based on ideal body weight. The 200mg tablet is therefore not suitable for use in children with an ideal body weight of less than 31kg.

4.3 Contraindications

- known hypersensitivity to 4-aminoquinoline compounds
- pre-existing maculopathy of the eye
- below 6 years of age (200mg tablets not adapted for weight <35kg) or for ideal body weight < 31 kg (see section 4.2)

4.4 Special warnings and precautions for use

Retinopathy

- All patients should have an ophthalmological examination before treatment with Plaquenil is initiated. Thereafter, ophthalmological examinations must be repeated at least every 12 months.
- Retinal toxicity is largely dose-related. The risk of retinal damage is small with daily doses of up to 6.5 mg/kg body weight. Exceeding the recommended dose sharply increases the risk of retinal toxicity.

The examination should include testing visual acuity and colour vision, careful ophthalmoscopy, fundoscopy and central visual field testing with a red target.

This examination should be more frequent and adapted to the patient in the following situations:

- daily dosage exceeds 6.5mg/kg lean body weight. Absolute body weight used as a guide to dosage could result in an overdosage in the obese.
- renal insufficiency
- visual acuity below 6/8
- age above 65 years
- cumulative dose more than 200 g.

Plaquenil should be discontinued immediately in any patient who develops a pigmentary abnormality, visual field defect or any other abnormalities not explained by difficulty in accommodation (see also section 4.8). Patients should continue to be observed as retinal changes and visual disturbances may progress even after cessation of therapy (see also section 4.8).

Concomitant use of hydroxychloroquine with drugs known to induce retinal toxicity, such as tamoxifen, is not recommended

Hypoglycaemia

Hydroxychloroquine has been shown to cause severe hypoglycaemia including loss of consciousness that could be life threatening in patients treated with and without antidiabetic medications. Patients treated with hydroxychloroquine should be warned about the risk of hypoglycaemia and the associated clinical signs and symptoms. Patients presenting with clinical symptoms suggestive of hypoglycaemia during treatment with hydroxychloroquine should have their blood glucose level checked and treatment reviewed as necessary.

QT interval prolongation

Hydroxychloroquine has potential to prolong the QTc interval in patients with specific risks factors. Hydroxychloroquine should be used with caution in patients with congenital or documented acquired QT prolongation and/or known risk factors for prolongation of the QT interval such as:

- cardiac disease, e.g., heart failure, myocardial infarction
- proarrhythmic conditions, e.g., bradycardia (< 50 bpm)
- a history of ventricular dysrhythmias
- uncorrected hypokalemia and/or hypomagnesemia
- during concomitant administration with QT interval prolonging agents (see section 4.5) as this may lead to an increased risk for ventricular arrhythmias.

The magnitude of QT prolongation may increase with increasing concentrations of the drug. Therefore, the recommended dose should not be exceeded (see also sections 4.5 and 4.8).

If signs of cardiac arrhythmia occur during treatment with hydroxychloroquine, treatment should be stopped and an ECG should be performed.

Chronic cardiac toxicity
Cases of cardiomyopathy resulting in cardiac failure, in some cases with fatal outcome, have been reported in patients treated with Plaquenil (see Section 4.8 and Section 4.9). Clinical monitoring for signs and symptoms of cardiomyopathy is advised. and Plaquenil should be discontinued if cardiomyopathy develops.

Chronic toxicity should be considered when conduction disorders (bundle branch block / atrio-ventricular heart block) as well as biventricular hypertrophy are diagnosed (see Section 4.8).

Plaquenil should be used with caution in patients taking medicines which may cause adverse ocular or skin reactions. Caution should also be applied when it is used in the following:

- patients with hepatic or renal disease, and in those taking medicines known to affect those organs. Estimation of plasma hydroxychloroquine levels should be undertaken in patients with severely compromised renal or hepatic function, and dosage adjusted accordingly.
- patients with severe gastrointestinal, neurological or blood disorders.

Caution is also advised in patients with a sensitivity to quinine, those with glucose-6-phosphate dehydrogenase deficiency, those with porphyria cutanea tarda which can be exacerbated by hydroxychloroquine, and in patients with psoriasis since it appears to increase the risk of skin reactions.

Suicidal behaviour has been reported in very rare cases in patients treated with hydroxychloroquine.

Small children are particularly sensitive to the toxic effects of 4-aminoquinolines; therefore, patients should be warned to keep Plaquenil out of the reach of children.

Other monitoring on long-term treatments
Patients on long term therapy should have periodic full blood counts, and hydroxychloroquine should be discontinued if abnormalities develop (see section 4.8).
All patients on long-term therapy should undergo periodic examination of skeletal muscle function and tendon reflexes. If weakness occurs, the drug should be withdrawn (see section 4.8).

Potential carcinogenic risk
Experimental data showed a potential risk of inducing gene mutations. Animal carcinogenicity data is only available for one species for the parent drug chloroquine and this study was negative (see section 5.3). In humans, there are insufficient data to rule out an increased risk of cancer in patients receiving-long term treatment.

Patients with Rare hereditary problems of galactose intolerance, the Lapp lactase deficiency or glucose-galactose malabsorption should not take this medicine.

Extrapyramidal disorders may occur with Plaquenil (See Section 4.8).

4.5 Interactions with other medicinal products and other forms of interaction

Hydroxychloroquine sulphate has been reported to increase plasma digoxin levels. Serum digoxin levels should be closely monitored in patients receiving concomitant treatment.

Hydroxychloroquine sulphate may also be subject to several of the known interactions of chloroquine even though specific reports have not appeared. These include: potentiation of its direct blocking action at the neuromuscular junction by aminoglycoside antibiotics; inhibition of its metabolism by cimetidine which may increase plasma concentration of the antimalarial; antagonism of effect of neostigmine and

pyridostigmine; reduction of the antibody response to primary immunisation with intradermal human diploid-cell rabies vaccine.

Drugs known to prolong QT interval / with potential to induce cardiac arrhythmia
Hydroxychloroquine should be used with caution in patients receiving drugs known to prolong the QT interval, e.g., Class IA and III antiarrhythmics, tricyclic antidepressants, antipsychotics, some anti-infectives due to increased risk of ventricular arrhythmia (see sections 4.4 and 4.9). Halofantrine should not be administered with hydroxychloroquine.

An increased plasma ciclosporin level was reported when ciclosporin and hydroxychloroquine were co-administered.

Administration of hydroxychloroquine with antimalarials known to lower the convulsion threshold (e.g mefloquine) may increase the risk of convulsions.

The activity of antiepileptic drugs might be impaired if co-administered with hydroxychloroquine.

As with chloroquine, antacids may reduce absorption of hydroxychloroquine so it is advised that a four hour interval be observed between Plaquenil and antacid dosaging.

In a single-dose interaction study, chloroquine has been reported to reduce the bioavailability of praziquantel. It is not known if there is a similar effect when hydroxychloroquine and praziquantel are coadministered. Per extrapolation, due to the similarities in structure and pharmacokinetic parameters between hydroxychloroquine and chloroquine, a similar effect may be expected for hydroxychloroquine.

There is a theoretical risk of inhibition of intra-cellular α-galactosidase activity when hydroxychloroquine is co-administered with agalsidase.

Concurrent use with drugs with oculotoxic or haemotoxic potential should be avoided if possible.

As hydroxychloroquine may enhance the effects of a hypoglycaemic treatment, a decrease in doses of insulin or antidiabetic drugs may be required.

4.6 Fertility, pregnancy and

lactation Pregnancy:

Only limited non-clinical data are available for hydroxychloroquine. In animal studies, reproduction toxicity was found with chloroquine, a substance related to hydroxychloroquine, following high maternal exposure. Chloroquine preclinical data show a potential risk of genotoxicity in some test systems (see section 5.3)

For hydroxychloroquine, when used on long-term therapy with high dosages for auto-immune diseases: Observational studies, as well as a meta-analysis including prospective studies in long-term use with large exposure have not observed a statistically significant increased risk of congenital malformations or poor pregnancy outcomes.

Hydroxychloroquine crosses the placenta. It should be noted that 4-aminoquinolines in therapeutic doses have been associated with central nervous system damage, including ototoxicity (auditory and vestibular toxicity, congenital deafness), retinal hemorrhages and abnormal retinal pigmentation. These effects were not confirmed in larger series/observational studies. Observational studies, as well as a meta-analysis including prospective studies in long-term use with large exposure have not observed a statistically significant increased risk of congenital malformations or poor pregnancy outcomes

Therefore hydroxychloroquine sulfate should be avoided in pregnancy except when, in the judgement of the physician, the individual potential benefits outweigh the potential hazards.

Fertility

There is no information available on the effect of Hydroxychloroquine sulfate on human fertility. In animal studies, chloroquine, a substance related to hydroxychloroquine, showed adverse effects on male fertility (see section 5.3).

Lactation:

Hydroxychloroquine is excreted in breast milk (less than 2% of the maternal dose after bodyweight correction). Careful consideration should be given to long term treatment with hydroxychloroquine during lactation because of the slow elimination rate and the potential for accumulation of a toxic amount in the infant. It is known that infants are extremely sensitive to the toxic effects of 4-aminoquinolines.
There are very limited data on the safety in the breastfed infant during hydroxychloroquine long- term treatment; the prescriber should assess the potential risks and benefits of use during breastfeeding, according to indication and duration of treatment.

4.7 Effects on ability to drive and use machines

Impaired visual accommodation soon after the start of treatment, which can cause blurring of vision, has been reported and patients should be warned regarding driving or operating machinery. If the condition is not self-limiting it will resolve on reducing the dose or stopping treatment.

4.8 Undesirable effects

The following CIOMS frequency rating is used, when applicable:
Very common (≥1/10), Common (≥1/100 to <1/10), Uncommon (≥1/1,000 to <1/100), Rare (≥1/10,000 to <1/1,000), Very rare (<1/10,000), not known (cannot be estimated from the available data).

	Very common	*Common*	*Uncommon*	*Rare*	*Very rare*	*Not known*
Blood and lymphatic system disorders						Bone marrow depression, anemia, aplastic anemia, agranulocytosis, leucopenia, thrombocytopenia
Immune system disorders						Urticaria, angioedema, bronchospasm
Metabolism and nutrition disorders		Anorexia				Hypoglycemia Hydroxychloroquine may exacerbate porphyria
Psychiatric disorders		Affect lability	Nervousness			Psychosis, suicidal

	Very common	*Common*	*Uncommon*	*Rare*	*Very rare*	*Not known*
Nervous system disorders		Headache	Dizziness			Convulsions have been reported with this class of drugs. Extrapyramidal disorders such as dystonia, dyskinesia,
Eye disorders		Blurring of vision due to a disturbanc e of accommo d ation which is dose dependent and reversible	Retinopathy, with changes in pigmentation and visual field defects. In its early form, it appears reversible on discontinuation of hydroxychloroquine. If allowed to develop, there may be a risk of progression even after treatment withdrawal. Patients with retinal changes may be asymptomatic initially, or may have scotomatous vision with paracentral, pericentral ring types, temporal scotomas and abnormal colour vision. Corneal changes including edema and opacities have been reported. They are either symptomless or may cause disturbances such as halos, blurring of vision, or photophobia.			Cases of maculopathies and macular degeneration have been reported and may be irreversible.
Ear and labyrinth disorders			Vertigo, tinnitus			Hearing loss
Cardiac disorders						QT interval prolongation in patients with specific risk factors, which may lead to arrhythmia (torsade de pointes, ventricular tachycardia) Cardiomyopath y which may result in

	Very common	*Common*	*Uncommon*	*Rare*	*Very rare*	*Not known*
						and in some cases a fatal outcome (see Section 4.4 and Section 4.9). Chronic toxicity should be considered when conduction disorders (bundle branch block / atrio-ventricular heart block) as well as biventricular hypertrophy
Gastrointe stinal disorders	Abdomi n al pain, nausea	Diarrhoea, vomiting These symptoms usually resolve immediate l y on reducing the dose or on stopping the				
Hepatobil i ary disorders			Abnormal liver function tests			Fulminant hepatic failure
Skin and subcutane o us tissue disorders		Skin rash, pruritus	Pigmentation disorders in skin and mucous membranes, bleaching of hair, alopecia These usually resolve readily on stopping treatment.			Bullous eruptions including erythema multiforme, Stevens-Johnson syndrome, and toxic epidermal necrolysis, Drug Rash with Eosinophilia and Systemic Symptoms (DRESS syndrome), photosensitivit y, exfoliative dermatitis, acute

	Very common	*Common*	*Uncommon*	*Rare*	*Very rare*	*Not known*
						(AGEP). AGEP has to be distinguished from psoriasis, although hydroxychloroqu i ne may precipitate attacks of psoriasis. It may be associated with fever and hyperleukocytos is . Outcome is usually
Musculoskeletal and connective tissue disorders			Sensorimotor disorders			Skeletal muscle myopathy or neuromyopathy leading to progressive weakness and atrophy of proximal muscle groups. Myopathy may be reversible after drug discontinuation, but recovery may take many months. Depression of tendon reflexes and abnormal nerve

Reporting of suspected adverse reactions

Reporting suspected adverse reactions after authorisation of the medicinal product is important. It allows continued monitoring of the benefit/risk balance of the medicinal product. Healthcare professionals are asked to report any suspected adverse reactions via HPRA Pharmacovigilance, Website: www.hpra.ie.**4.9**

Overdose

Overdosage with the 4-aminoquinolines is dangerous particularly in infants, as little as 1-2g having proved fatal.

The symptoms of overdosage may include headache, visual disturbances, cardiovascular collapse, convulsions, hypokalaemia, rhythm and conduction disorders, including QT prolongation, torsade de pointes, ventricular tachycardia and ventricular fibrillation, width-increased QRS complex, bradyarrhythmias, nodal rhythm, atrioventricular block,, followed by sudden and potentially fatal respiratory and cardiac arrest. Immediate medical attention is required, as these effects may appear shortly after the overdose. The stomach should be immediately evacuated, either by emesis or by gastric lavage. Activated charcoal in a dose at least five times that of the overdosage may inhibit further absorption if introduced into the stomach by tube, following lavage, and within 30 minutes of ingestion of the overdose.

Consideration should be given to administration of parenteral diazepam in cases of overdosage; it has been shown to be beneficial in reversing chloroquine cardiotoxicity.

Respiratory support and shock management should be instituted as necessary.

5. PHARMACOLOGICAL PROPERTIES

5.1 Pharmacodynamic properties

Antimalarial agents like chloroquine and hydroxychloroquine have several pharmacological actions which may be involved in their therapeutic effect in the treatment of rheumatic disease, but the role of each is not known. These include interaction with sulphydryl groups, interference with enzyme activity (including phospholipase, NADH - cytochrome C reductase, cholinesterase, proteases and hydrolases), DNA binding, stabilisation of lysosomal membranes, inhibition of prostaglandin formation, inhibition of polymorphonuclear cell chemotaxis and phagocytosis, possible interference with interleukin 1 production from monocytes and inhibition of neutrophil superoxide release.

5.2 Pharmacokinetic properties

Hydroxychloroquine is rapidly absorbed following oral administration. Mean bioavailability is approximately 74%. It is widely distributed throughout the body, accumulating within blood cells and other tissues such as liver, lungs, kidneys and eyes. It is partially converted to active ethylated metabolites in the liver and eliminated principally via the kidney, 23 to 25% unchanged, but also via the bile. Excretion is slow, the terminal elimination half-life being approximately 50 days (whole blood) and 32 days (plasma).

Hydroxychloroquine crosses the placenta and is likely to resemble chloroquine in entering breast milk.

5.3 Preclinical safety data

Only limited preclinical data are available for hydroxychloroquine, therefore chloroquine data are considered due to the similarity of structure and pharmacological properties between the 2 products.

Genotoxicity/Carcinogenicity
There are limited data on hydroxychloroquine genotoxicity or carcinogenicity. Chloroquine, a substance related to hydroxychloroquine was genotoxic in non-GLP in-vitro tests. A non-GLP 2-year dietary administration carcinogenicity study of chloroquine in rats did not show any carcinogenic potential.
Reproductive and developmental toxicity
Hydroxychloroquine crosses the placenta. In non-GLP studies with mice and monkeys, transplacental transfer chloroquine, a substance related to hydroxychloroquine, was demonstrated with accumulation in foetal eye and ear tissue. High maternal doses of chloroquine were foetotoxic in rats and caused anophthalmia and microophthalmia. In studies in rats, chloroquine reduced the testosterone secretion, the weight of the testis and epidedymis and caused production of abnormal sperm.

There are no preclinical safety data of relevance to the prescriber, which are additional to that already included in other sections of the SPC.

6. PHARMACEUTICAL PARTICULARS

6.1 List of excipients

Lactose Monohydrate
Maize starch

Magnesiu
m stearate
Povidone
Opadry OY-L-28900 (containing Hypromellose, Macrogol 4000, Titanium dioxide (E171), Lactose monohydrate).

6.2 Incompatibilities

Not applicable.

6.3 Shelf life

3 years.

6.4 Special precautions for storage

This medicinal product does not require any special storage conditions

6.5 Nature and contents of container

i) Amber glass bottle with tin plate screw cap. Pack size 100 tablets.
ii) HDPE bottle with LDPE cap. Pack size 56 tablets.
iii) PVC/aluminium foil blister pack. Pack size 56 or 60 tablets.

Not all pack sizes may be marketed.

6.6 Special precautions for disposal

No special requirements

7. MARKETING AUTHORISATION HOLDER

sanofi-aventis Ireland Limited T/A
SANOFI Citywest Business
Campus
Dubline 24
Ireland

8. MARKETING AUTHORISATION NUMBER

PA 540/155/1

9. DATE OF FIRST AUTHORISATION/RENEWAL OF AUTHORISATION

Date of first authorisation: 1st April

1977 Date of last renewal: 1st April

2007

10. DATE OF (PARTIAL) REVISION OF TEXT

March 2020

Appendix B – Azithromycin Summary of Products Characteristics

Summary of Product Characteristics

1 NAME OF THE MEDICINAL PRODUCT

Azithromycin Clonmel 250 mg film-coated tablets.

2 QUALITATIVE AND QUANTITATIVE COMPOSITION

1 film-coated tablet contains azithromycin monohydrate equivalent to 250 mg azithromycin.
Excipients with known effect
Contains Soya Lecithin.

For the full list of excipients, see section 6.1.

3 PHARMACEUTICAL FORM

Film-coated tablet.

White to off-white, oblong, film-coated, plain on both sides.

4 CLINICAL PARTICULARS

4.1 Therapeutic Indications

Azithromycin Clonmel is indicated for the treatment of the following infections, when caused by micro-organisms sensitive to azithromycin (see 4.4 and 5.1):

- acute bacterial sinusitis (adequately diagnosed)
- acute bacterial otitis media (adequately diagnosed)
- pharyngitis, tonsillitis
- acute exacerbation of chronic bronchitis (adequately diagnosed)
- mild to moderate severe community acquired pneumonia
- skin and soft tissue infections
- uncomplicated Chlamydia trachomatis urethritis and cervicitis

Considerations should be given to official guidance on the appropriate use of antibacterial agents.
Azithromycin is not the first choice for the empiric treatment of infections in areas where the prevalence of resistant isolates is 10% or more.

4.2 Posology and method of administration

Posology

Adults
In uncomplicated *Chlamydia trachomatis* urethritis and cervicitis the dosage is 1,000 mg as a single oral dose.
For all other indications the dose is 1,500 mg, to be administered as 500 mg per day for three consecutive days. As an alternative the same total dose (1,500 mg) can also be administered

over a period of five days with 500 mg on the first day and 250 mg on the second to the fifth day.

Elderly
The same dosage as in adult patients is used in the elderly. Since elderly patients can be patients with ongoing proarrhythmic conditions a particular caution is recommended due to the risk of developing cardiac arrhythmia and torsades de pointes (see section 4.4).

Renal impairment
No dose adjustment is necessary in patients with mild to moderate renal impairment (GFR 10-80 ml/min) (see section 4.4).

Hepatic impairment
A dose adjustment is not necessary for patients with mild to moderately impaired liver function (see section 4.4).

Paediatric population
Azithromycin Clonmel tablets should only be administered to children weighing more than 45 kg when normal adult dose should be used. For children under 45 kg other pharmaceutical forms of azithromycin, e.g. suspensions, may be used.

Method of administration For oral use.
Azithromycin Clonmel should be given as a single daily dose. The tablets may be taken with food.

3.1 Contraindications

Hypersensitivity to the active substance, to erythromycin, any macrolide or ketolide antibiotic, soya, peanut or to any of the excipients listed in Section 6.1.

3.2 Special warnings and precautions for use

As with erythromycin and other macrolides, serious allergic reactions including angioneurotic oedema and anaphylaxis (rarely fatal), dermatologic reactions including acute generalised exanthematous pustulosis (AGEP), Stevens-Johnson syndrome (SJS), toxic epidermal necrolysis (TEN) (rarely fatal) and Drug Reaction with Eosinophilia and Systemic Symptoms (DRESS) have been reported. Some of these reactions with azithromycin have resulted in recurrent symptoms and required a longer period of observation and treatment.

If an allergic reaction occurs, the drug should be discontinued and appropriate therapy should be instituted. Physicians should be aware that reappearance of the allergic symptoms may occur when symptomatic therapy is discontinued.

Since liver is the principal route of elimination for azithromycin, the use of azithromycin should be undertaken with caution in patients with significant hepatic disease. Cases of fulminant hepatitis potentially leading to life-threatening liver failure have been reported with azithromycin (see Section 4.8). Some patients may have had pre-existing hepatic disease or may have been taking other hepatotoxic medicinal products.

In case of signs and symptoms of liver dysfunction, such as rapid developing asthenia associated with jaundice, dark urine, bleeding tendency or hepatic encephalopathy, liver function tests/ investigations should be performed immediately. Azithromycin administration should be stopped if liver dysfunction has emerged.

In patients receiving ergot derivatives, ergotism has been precipitated by co-administration of some macrolide antibiotics. There are no data concerning the possibility of an interaction between ergot and azithromycin. However, because of the theoretical possibility of ergotism, azithromycin and ergot derivatives should not be co-administered.

As with any antibiotic preparation, observation for signs of superinfection with non-susceptible organisms, including fungi is recommended.
Clostridium difficile associated diarrhoea (CDAD) has been reported with use of nearly all antibacterial agents, including azithromycin, and may range in severity from mild diarrhoea to fatal colitis. Treatment with antibacterial agents alters the normal flora of the colon leading to overgrowth of *C. difficile*.

C. difficile produces toxins A and B which contribute to the development of CDAD. Hypertoxin producing strains of *C. difficile* cause increased morbidity and mortality, as these infections can be refractory to antimicrobial therapy and may require colectomy. CDAD must be considered in all patients who present with diarrhoea following antibiotic use. Careful medical history is necessary since CDAD has been reported to occur over two months after the administration of antibacterial agents.
Pseudomembranous colitis has been reported with the use of macrolide antibiotics. This diagnosis should therefore be considered in patients who get diarrhoea after starting the treatment with azithromycin. Should pseudomembranous colitis be induced by azithromycin, then anti-peristaltics should be contraindicated.

There is no experience regarding the safety and efficacy of the long-term application of azithromycin for the above mentioned indications. In case of quickly recurring infections, treatment with an other antibacterial agent should be considered.

Use in renal impairment: No dose adjustment is necessary in patients with mild to moderate renal impairment (GFR 10–80 ml/min). Caution is advised in patients with severe renal impairment (GFR < 10 ml/min) a 33 % increase in systemic exposure to azithromycin was observed (see section 5.2).

Prolonged cardiac repolarisation and QT interval, imparting a risk of developing cardiac arrhythmia and torsades de pointes, have been seen in treatment with other macrolides including azithromycin (see section 4.8). Therefore as the following situations may lead to an increased risk for ventricular arrhythmias (including torsade de pointes) which can lead to cardiac arrest, azithromycin should be used with caution in patients with ongoing proarrhythmic conditions (especially women and elderly patients) such as patients:

- With congenital or documented acquired QT prolongation.
- Currently receiving treatment with other active substances known to prolong QT interval such as antiarrhythmics of class IA (quinidine and procainamide) and class III (dofetilide, amiodarone and sotalol), cisapride and terfenadine; antipsychotic agents such as pimozide; antidepressants such as citalopram; and fluoroquinolones such as moxifloxacin and levofloxacin.

- With electrolyte disturbance, particularly in cases of hypokalaemia and hypomagnesaemia
- With clinically relevant bradycardia, cardiac arrhythmia or severe cardiac insufficiency.

Exacerbations of the symptoms of myasthenia gravis and new onset of myasthenia syndrome have been reported in patients receiving azithromycin therapy (See Section 4.8).

Safety and efficacy for the prevention or treatment of MAC (Mycobacterium Avium Complex) in children have not been established.

Azithromycin is not the substance of first choice for the treatment of pharyngitis and tonsillitis caused by *Streptococcus pyogenes*. For this and for the prophylaxis of acute rheumatic fever penicillin is the treatment of first choice.

Pneumonia
Due to the emerging resistance of *Streptococcus pneumoniae* towards macrolides azithromycin is not the drug of first choice in community acquired pneumonia. In hospital acquired pneumonia azithromycin should only be used in combination with further appropriate antibiotics.

Skin and soft tissue infections
The main causative agent of soft tissue infections, Staphylococcus aureus, is frequently resistant to azithromycin. Therefore, susceptibility testing is considered a precondition for treatment of soft tissue infections with azithromycin.

Sinusitis
Often, azithromycin is not the substance of first choice for the treatment of sinusitis.

Acute otitis media
Often, azithromycin is not the substance of first choice for the treatment of acute otitis media.

In case of sexually transmitted diseases a concomitant infection by *T. pallidum* should be excluded. Azithromycin should be administered with caution to patients with neurological or psychiatric disorders.
It is recommended that prothrombin time be monitored in patients receiving concomitant treatment with anticoagulants (see section 4.5).
Azithromycin is not indicated for the treatment of infected burn wounds.

Azithromycin film-coated tablets are not suitable for treatment of severe infections where a high concentration of the antibiotic in the blood is rapidly needed.

Due to cross-resistance existing among macrolides, in areas with a high incidence of erythromycin resistance, it is especially important to take into consideration the evolution of the pattern of susceptibility to azithromycin and other antibiotics (see section 5.1).

Long term use
There is no experience regarding the safety and efficacy of long term use of azithromycin for the mentioned indications. In case of rapid recurrent infections, treatment with another antibiotic should be considered.

3.1 Interaction with other medicinal products and other forms of interaction

Antacids

In a pharmacokinetic study on the effect of concomitant administration of antacids and azithromycin, no effect on the total bio-availability was seen, although the peak serum levels were reduced by approximately 25 %. In patients receiving both azithromycin and antacids, the drugs should not be taken simultaneously. Azithromycin should be taken at least 1 hour before or 2 hours after the antacid.

Cetirizine: In healthy volunteers, co-administration of a 5-day regimen of azithromycin with cetirizine 20 mg at steady- state resulted in no pharmacokinetic interaction and no significant changes in the QT interval.

Didanosine (Dideoxyinosine): Co-administration of 1,200 mg/day azithromycin with 400 mg/day didanosine in 6 HIV- positive subjects did not appear to affect the steady-state pharmacokinetics of didanosine as compared with placebo.

Digoxin and colchicine

Concomitant administration of macrolide antibiotics, including azithromycin, with P-glycoprotein substrates such as digoxin and colchicine, has been reported to result in increased serum levels of the P-glycoprotein substrate. Therefore, if azithromycin and P-glycoprotein substrates such as digoxin are administered concomitantly, the possibility of elevated serum digoxin concentrations should be considered. Clinical monitoring, and possibly serum digoxin levels, during treatment with azithromycin and after its discontinuation are necessary.

Zidovudine

Single administrations of 1,000 mg of azithromycin and multiple administrations of 600 mg or 1,200 mg azithromycin had no effect on the plasma pharmacokinetics or the renal excretion of zidovudine or its glucuronide metabolite.

However, administration of azithromycin increased the concentrations of phosphorylated zidovudine, the clinically active metabolite, in peripheral blood mononuclear cells. The clinical significance of this finding is unclear, but it may be of benefit to patients.

Azithromycin does not interact significantly with the hepatic cytochrome P450 system. It is not believed to undergo the pharmacokinetic drug interactions as seen with erythromycin and other macrolides. Hepatic cytochrome P450 induction or inactivation via cytochrome-metabolite complex does not occur with azithromycin.

Ergotamine derivatives

In patients treated with ergotamine derivatives ergotism can be induced by the concomitant administration of some macrolide antibiotics. There is no known data about the possibility of an interaction between ergotamine derivatives and azithromycin. Because of the theoretical possibility of ergotism azithromycin and ergotamine derivatives should not be combined.

Pharmacokinetic studies have been conducted between azithromycin and the following drugs known to undergo significant cytochrome P450 mediated metabolism.

Atorvastatin: Co-administration of atorvastatin (10 mg daily) and azithromycin (500 mg daily) did not alter the plasma concentrations of atorvastatin (based on a HMG CoA-

reductase inhibition assay). However, post-marketing cases of rhabdomyolysis in patients receiving azithromycin with statins have been reported.

Carbamazepine: In a pharmacokinetic interaction study in healthy volunteers, no significant effect was observed on the plasma levels of carbamazepine or its active metabolite in patients receiving concomitant azithromycin.

Cimetidine: In a pharmacokinetic study investigating the effects of a single dose of cimetidine, given 2 hours before azithromycin, on the pharmacokinetics of azithromycin, no alteration of azithromycin pharmacokinetics was seen.

Coumarin-Type Oral Anticoagulants: In a pharmacokinetic interaction study, azithromycin did not alter the anticoagulant effect of a single 15-mg dose of warfarin administered to healthy volunteers. There have been reports received in the post-marketing period of potentiated anticoagulation subsequent to co-administration of azithromycin and coumarin-type oral anticoagulants. Although a causal relationship has not been established, consideration should be given to the frequency of monitoring prothrombin time when azithromycin is used in patients receiving coumarin-type oral anticoagulants.

Cyclosporin: In a pharmacokinetic study with healthy volunteers that were administered a 500 mg/day oral dose of azithromycin for 3 days and were then administered a single 10 mg/kg oral dose of cyclosporin, the resulting cyclosporin Cmax and AUC0-5 were found to be significantly elevated. Consequently, caution should be exercised before considering concurrent administration of these drugs. If co-administration of these drugs is necessary, cyclosporin levels should be monitored and the dose adjusted accordingly.

Efavirenz: Co-administration of a 600 mg single dose of azithromycin and 400 mg efavirenz daily for 7 days did not result in any clinically significant pharmacokinetic interactions.

Fluconazole

Co-administration of a single dose of 1,200 mg azithromycin did not alter the pharmacokinetics of a single dose of 800 mg Fluconazole. Total exposure and half-life of azithromycin were unchanged by the co-administration of fluconazole, however, a clinically insignificant decrease in Cmax (18 %) of azithromycin was observed.

Indinavir: Co-administration of a single dose of 1,200 mg azithromycin had no statistically significant effect on the pharmacokinetics of indinavir administered as 800 mg three times daily for 5 days.

Methylprednisolone: In a pharmacokinetic interaction study in healthy volunteers, azithromycin had no significant effect on the pharmacokinetics of methylprednisolone.

Midazolam: In healthy volunteers, co-administration of azithromycin 500 mg/day for 3 days did not cause clinically significant changes in the pharmacokinetics and pharmacodynamics of a single 15 mg dose of midazolam.

Nelfinavir: Co-administration of azithromycin (1,200 mg) and nelfinavir at steady state (750 mg three times daily) resulted in increased azithromycin concentrations. No clinically significant adverse effects were observed and no dose adjustment is required.

Rifabutin

Co-administration of azithromycin and rifabutin did not affect the serum concentrations of either active substance. Neutropenia was observed in subjects receiving concomitant treatment of azithromycin and rifabutin. Although neutropenia has been associated with the use of rifabutin, a causal relationship to combination with azithromycin has not been established (see section 4.8).

Sildenafil: In normal healthy male volunteers, there was no evidence of an effect of azithromycin (500mg daily for 3 days) on the AUC and Cmax, of sildenafil or its major circulating metabolite.
Terfenadine: Pharmacokinetic studies have reported no evidence of an interaction between azithromycin and terfenadine. There have been rare cases reported where the possibility of such an interaction could not be entirely excluded; however there was no specific evidence that such an interaction had occurred.
Theophylline
Pharmacokinetic studies in healthy volunteers revealed no interaction between azithromycin and theophylline with concomitant administration. Since interactions of other macrolides with theophylline were reported, care should be taken of signs of increased theophylline levels.
Triazolam: In 14 healthy volunteers, co-administration of azithromycin 500 mg on Day 1 and 250 mg on Day 2 with
0.125 mg triazolam on Day 2 had no significant effect on any of the pharmacokinetic variables for triazolam compared to triazolam and placebo.

Trimethoprim/sulfamethoxazole
Co-administration of trimethoprim/sulfamethoxazole (160 mg/800 mg) for 7 days with azithromycin 1,200 mg on day 7 had no significant effect on peak concentrations, total exposure or urinary excretion of either trimethoprim or sulfamethoxazole. Azithromycin serum concentrations were similar to those seen in other studies.

Cisapride
Cisapride is metabolised in the liver by the enzyme CYP 3A4. Because macrolides inhibit this enzyme, concomitant administration of cisapride may cause the increase of QT interval prolongation, ventricular arrhythmias and torsades de pointes.

Astemizole, alfentanil
There is no known data regarding interaction with astemizole or alfentanil. Caution is needed in the concomitant use of these medicinal products and azithromycin, as an increase of action with the concomitant use of the macrolide antibiotic erythromycin has been described.

Protease Inhibitors
Co-administration of a single dose of 1,200 mg azithromycin had no statistically significant effect on the pharmacokinetics of indinavir administered as 800 mg three times daily for 5 days.

4.6 Fertility, pregnancy and lactation
Pregnancy
There are no adequate data from the use of azithromycin in pregnant women. In reproduction toxicity studies in animals azithromycin was shown to pass the placenta, but no teratogenic effects were observed. The safety of azithromycin has not been confirmed with regard to the

use of the active substance during pregnancy. Therefore azithromycin should only be used during pregnancy if the benefit outweighs the risk.

Breast-feeding
Azithromycin has been reported to be secreted into human breast milk, but there are no adequate and well-controlled clinical studies in nursing women that have characterized the pharmacokinetics of azithromycin excretion into human breast milk. Azithromycin should not be used in the treatment of a lactating woman unless the physician feels that the potential benefits justify the potential risks to the infant.

Fertility
In fertility studies conducted in rat, reduced pregnancy rates were noted following administration of azithromycin. The relevance of this finding to humans is unknown.

4.7 Effects on ability to drive and use machines

No studies on the effects on the ability to drive and use machines have been performed. However, the possibility of undesirable effects like dizziness and convulsions should be taken into account when performing these activities.

4.8 Undesirable effects

The table below lists the adverse reactions identified through clinical trial experience and post-marketing surveillance by system organ class and frequency. Adverse reactions identified from post-marketing experience are included in italics. The frequency grouping is defined using the following convention: Very common (2' 1/10); Common (2' 1/100 to < 1/10); Uncommon (2' 1/1,000 to < 1/100); Rare (2' 1/10,000 to < 1/1,000); Very Rare (< 1/10,000); and Not known (cannot be estimated from the available data). Within each frequency grouping, undesirable effects are presented in order of decreasing seriousness.

Adverse reactions possibly or probably related to azithromycin based on clinical trial experience and post- marketing surveillance:

System Organ Class	Very Common	Common	Uncommon	Rare	Very Rare	Not Known
**Infection and Infestations**			Candidiasis, oral candidiasis, vaginal infection, pneumonia, fungal infection, bacterial infection, pharyngitis, gastroenteritis, respiratory disorder, rhinitis			Pseudomembranous colitis (see section 4.4)
**Blood and Lymphatic System Disorders**			Leukopenia, neutropenia, eosinophilia			Thrombocytopenia, haemolytic anaemia
**Immune System Disorders**			Angioedema, hypersensitivity			Anaphylactic reaction (see section 4.4)

**Metabolism and Nutrition Disorders**		Anorexia				
**Psychiatric Disorders**			Nervousness	Agitation		Aggression, anxiety, delirium, hallucination
**Nervous System Disorders**		Dizziness, headache, paraesthesia, dysgeusia	Hypoaesthesia, somnolence, insomnia			Syncope, convulsion, psychomotor hyperactivity, anosmia, ageusia, parosmia, myasthenia gravis (see Section 4.4)
**Eye Disorders**		Visual impairment				
**Ear and Labyrinth Disorders**		Deafness	Hearing impaired, tinnitus, vertigo, ear disorder			
**Cardiac Disorders**			Palpitations			Torsades de pointes (see section 4.4), arrhythmia (see section 4.4) including ventricular tachycardia, Electrocardiogram QT prolonged (see section 4.4)
**Vascular Disorders**			Hot flush			Hypotension
**Respiratory, thoracic and mediastinal disorders**			Dyspnoea, epistaxis			
**Gastrointestinal Disorders**	Diarrhoea, abdominal pain, nausea, flatulence	Vomiting, dyspepsia	Gastritis, constipation, dysphagia, abdominal distension, dry mouth, eructation, mouth ulceration, salivary hypersecretion			Pancreatitis, tongue discolouration
**Hepatobiliary Disorders**			Hepatitis	Hepatic function abnormal, jaundice cholestatic		Hepatic failure (see section 4.4)**, hepatitis fulminant, hepatic necrosis
**Skin and Subcutaneous Tissue Disorders**		Rash, pruritus	Stevens-Johnson syndrome, photosensitivity reaction, urticaria, dermatitis, dry skin, hyperhidrosis	Acute generalised exanthematous pustulosis (AGEP)	DRESS-syndrome (Drug Reaction with Eosinophilia and Systemic Symptoms)	Toxic epidermal necrolysis, erythema multiforme
**Musculoskeletal and Connective**		Arthralgia	Osteoarthritis, myalgia, back pain, neck pain			

**Tissue Disorders**						
**Renal and Urinary Disorders**			Dysuria, renal pain			Renal failure acute, nephritis interstitial
**Reproductive system and breast disorders**			Metrorrhagia, testicular disorder			
**General Disorders and Administration Site Conditions**		Injection site pain ,* injection site inflammation, * fatigue	Chest pain, oedema, malaise, asthenia, face oedema, pyrexia, pain, peripheral oedema			
**Investigations**		Lymphocyte count decreased, eosinophil count increased, blood bicarbonate decreased, basophils increased, monocytes increased, neutrophils increased	Aspartate aminotransferase increased, alanine aminotransferase increased, blood bilirubin increased, blood urea increased, blood creatinine increased, blood potassium abnormal, blood alkaline phosphatase increased, chloride increased, glucose increased, platelets increased, hematocrit decreased, bicarbonate increased, abnormal sodium			
**Injury and poisoning**			Post procedural complication			

* for powder for solution for infusion only
**which has rarely resulted in death

Azithromycin Clonmel film-coated tablets contain soya lecithin, which can very rarely cause allergic reactions.
Adverse reactions possibly or probably related to Mycobacterium Avium Complex prophylaxis and treatment based on clinical trial experience and post-marketing surveillance. These adverse reactions differ from those reported with immediate release or the prolonged release formulations, either in kind or in frequency:

	Very Common (-1/10)	Common (-1/100 to < 1/10)	Uncommon (-1/1,000 to <1/100)
Metabolism and nutrition disorders		Anorexia	
Nervous system disorders		Dizziness, headache,	Hypoaesthesia

		paraesthesia, dysgeusia	
Eye disorders		Visual impairment	
Ear and lanrynth disorders		Deafness	Hearing impaired, tinnitus
Cardiac disorders			Palpitations
Gastrointestinal disorders	Diarrhoea abdominal pain, nausea, flatulence, abdominal discomfort, loose stools		
Hepatobiliary disorders			Hepatitis
Skin and subcutaneous disorders		Rash, pruritus	Stevens-Johnson syndrome, photosensitivity reaction
Musculoskeletal and connective tissue disorders		Arthralgia	
General disorders and administration site conditions		Fatigue	Asthenia, malaise

Reporting of suspected adverse reactions

Reporting suspected adverse reactions after authorisation of the medicinal product is important. It allows continued monitoring of the benefit/risk balance of the medicinal product. Healthcare professionals are asked to report any suspected adverse reactions via HPRA Pharmacovigilance, Earlsfort Terrace, IRL - Dublin 2; Tel: +353 1 6764971; Fax: +353 1 6762517. Website: www.hpra.ie; E-mail: medsafety@hpra.ie.

4.9 Overdose

Symptoms

Adverse events experienced in higher than recommended doses were similar to those seen at normal doses. Characteristic symptoms of an overdose of macrolide antibiotics were: reversible hearing loss, severe nausea, vomiting and diarrhoea.

Management

In case of an overdose lavage and general supporting measures are indicated.

5 PHARMACOLOGICAL PROPERTIES

5.1 Pharmacodynamic properties

Pharmacotherapeutic group: Antibacterials for systemic use, macrolides ATC Code: J01FA10

Mechanism of action

Azithromycin is an azalide, derived from the macrolide class of antibiotics. The mode of action of azithromycin is inhibition of protein synthesis in bacteria by binding to the 50s ribosomal subunit and preventing translocation of peptides. Azithromycin is usually bacteriostatic. However, in high concentrations, azithromycin may be bactericidal against selected microorganisms. Azithromycin is active against many Gram-positive and Gram-negative aerobic and anaerobic bacteria and bacterial pathogens such as *Mycobacterium avium* complex, *Mycoplasma* spp., *Borrelia burgdorferi, Chlamydia* spp. and *Campylobacter* spp. In addition, azithromycin has activity against protozoan microorganisms such as *Toxoplasma gondii*.

Mechanism of resistance

The two most frequently encountered mechanisms of resistance to macrolides, including azithromycin, are target modification (most often by methylation of 23S rRNA) and active efflux. The occurrence of these resistance mechanisms varies from species to species and, within a species, the frequency of resistance varies by geographical location.

The most important ribosomal modification that determines reduced binding of macrolides is post transcriptional (N_6) dimethylation of adenine at nucleotide A2058 (*E. coli* numbering system) of the 23S rRNA by methylases encoded by *erm* (erythromycin ribosome methylase) genes. Ribosomal modifications often determine cross resistance (MLS_B phenotype) to other classes of antibiotics whose ribosomal binding sites overlap that of the macrolides: the lincosamides (including clindamycin), and the streptogramins B (which include, for example, the quinupristin component of quinupristin/dalfopristin). Different *erm* genes are present in different bacterial species, in particular streptococci and staphylococci. Susceptibility to macrolides can also be affected by less frequently encountered mutational changes in nucleotides A2058 and A2059, and at some other positions of 23S rRNA, or in the large subunit ribosomal proteins L4 and L22.

Efflux pumps occur in a number of species, including Gram-negatives, such as *Haemophilus influenzae* (where they may determine intrinsically higher MICs) and staphylococci. In streptococci and enterococci, an efflux pump that recognizes 14- and 15-membered macrolides (which include, respectively, erythromycin and azithromycin) is encoded by *mef*(A) genes.

Breakpoints

Azithromycin susceptibility breakpoints for typical bacterial pathogens, as published by EUCAST[1] are:

**Organism**	**MIC[2] breakpoints (mg/l)**	
	**Susceptible (S)**	**Resistant (R)**
Staphylococcus spp.	:S 1	> 2
Streptococcus groups A, B, C and G	:S 0.25 mg/l	> 0.5 mg/l
Streptococcus pneumoniae	:S 0.25 mg/l	> 0.5 mg/l
Haemophilus influenzae	:S 0.12 mg/l	> 4 mg/l
Moraxella catarrhalis	:S 0.25 mg/l	> 0.5 mg/l
Neisseria gonorrhoeae	:S 0.25 mg/l	> 0.5 mg/l

[1] EUCAST = European Committee on Antimicrobial Susceptibility Testing;

[2] MIC = Minimal Inhibitory Concentration

Susceptibility

The prevalence of acquired resistance may vary geographically and with time for selected species and local information on resistance is desirable, particularly when treating severe infections. As necessary, expert advice should be sought when the local prevalence of resistance is such that the utility of the agent in at least some types of infections is questionable.

Table: Antibacterial spectrum of azithromycin

**Commonly susceptible species**
**Aerobic Gram-positive microorganisms**
Staphylococcus aureus<br>Meticillin-susceptible
Streptococcus pneumoniae<br>Penicillin-susceptible
Streptococcus pyogenes **(Group A)**
**Aerobic Gram-negative microorganisms**
Haemophilus influenzae<br>*Haemophilus parainfluenzae*
Legionella pneumophila
Moraxella catarrhalis
Neisseria gonorrhoeae
Pasteurella multocida
**Anaerobic microorganisms**
Clostridium perfringens
Fusobacterium spp.
Prevotella spp.
Porphyromonas spp.
**Other microorganisms**
Chlamydia trachomatis
**Species for which acquired resistance may be a problem**
**Aerobic Gram-positive microorganisms**
Streptococcus pneumoniae<br>Penicillin-intermediate<br>Penicillin-resistant
**Inherently resistant organisms**
**Aerobic Gram-positive microorganisms**
Enterococcus faecalis
Staphylococci **MRSA, MRSE***
**Anaerobic microorganisms**
Bacteroides fragilis group

* Meticillin-resistant staphylococci have a very high prevalence of acquired resistance to macrolides and have been placed here because they are rarely susceptible to azithromycin.

5.1 Pharmacokinetic properties

Absorption

After oral administration the bioavailability of azithromycin is approximately 37 %. Peak plasma levels are reached after 2-3 hours (C_{max} after a single dose of 500 mg orally was approximately 0.4 mg/l).

Distribution
Kinetic studies have shown markedly higher azithromycin levels in tissue than in plasma (up to 50 times the maximum observed concentration in plasma) indicating that the active substance is heavily tissue bound (steady state distribution volume of approximately 31 l/kg). Concentrations in target tissues such as lung, tonsil, and prostate exceed the MIC_{90} for likely pathogens after a single dose of 500 mg.

In experimental *in vitro* and *in vivo* studies azithromycin accumulates in the phagocytes, freeing is stimulated by active phagocytosis. In animal studies this process appeared to contribute to the accumulation of azithromycin in the tissue. In serum the protein binding of azithromycin is variable and depending on the serum concentration varies from 50 % in
0.05 mg/l to 12 % in 0.5 mg/l.

Elimination
Plasma terminal elimination half-life closely reflects the tissue depletion half-life of 2 to 4 days. About 12 % of an intravenously administered dose is excreted in the urine unchanged over a period of 3 days; the majority in the first 24 hours. Biliary excretion of azithromycin, predominantly in unchanged form, is a major route of elimination. The identified metabolites (formed by N- and O-demethylising, by hydroxylising of the desosamine and aglycone rings, and by the splitting of the cladinose conjugate) are microbiologically inactive.

After a 5 day treatment slightly higher (29 %) AUC values were seen in the elderly volunteers (> 65 years of age) compared to the younger volunteers (< 45 years of age). However these differences are not regarded as clinically relevant; therefore a dose adjustment is not recommended.

Pharmacokinetics in special populations

Renal insufficiency
Following a single oral dose of azithromycin 1 g, mean C_{max} and AUC_{0-120} increased by 5.1 % and 4.2 % respectively, in subjects with mild to moderate renal impairment (glomerular filtration rate of 10-80 ml/min) compared with normal renal function (GFR > 80 ml/min). In subjects with severe renal impairment, the mean C_{max} and AUC_{0-120} increased 61 % and 35 % respectively compared to normal.

Hepatic insufficiency
In patients with mild to moderate hepatic impairment, there is no evidence of a marked change in serum pharmacokinetics of azithromycin compared to normal hepatic function. In these patients, urinary recovery of azithromycin appears to increase perhaps to compensate for reduced hepatic clearance.

Elderly
The pharmacokinetics of azithromycin in elderly men was similar to that of young adults; however, in elderly women, although higher peak concentrations (increased by 30-50 %) were observed, no significant accumulation occurred.

Infants, toddlers, children and adolescents
Pharmacokinetics have been studied in children aged 4 months – 15 years taking capsules, granules or suspension. At 10 mg/kg on day 1 followed by 5 mg/kg on days 2-5, the C_{max} achieved is slightly lower than adults with 224 ug/l in children aged 0.6-5 years and after 3 days dosing and 383 ug/l in those aged 6-15 years. The $t_{1/2}$ of 36 h in the older children was within the expected range for adults.

5.3 Preclinical safety data

In high-dose animal studies, giving active substance concentrations 40 fold higher than those expected in clinical practice, azithromycin has been noted to cause reversible phospholipidosis, generally without discernible toxicological consequences. There is no evidence that this is of relevance to the normal use of azithromycin in humans.

Carcinogenic potential:
Long-term studies in animals have not been performed to evaluate carcinogenic potential.

Mutagenic potential:
Azithromycin has shown no mutagenic potential in standard laboratory tests: mouse lymphoma assay, human lymphocyte clastogenic assay, and mouse bone marrow clastogenic assay.

Reproductive toxicity:
No teratogenic effects were observed in animal studies of embryotoxicity in mice and rats. In rats, azithromycin dosages of 100 and 200 mg/kg bodyweight/day led to mild retardations in foetal ossification and in maternal weight gain. In peri-/postnatal studies in rats, mild retardations following treatment with 50 mg/kg/day azithromycin and above were observed.

6 PHARMACEUTICAL PARTICULARS

6.1 List of excipients

Core:
Microcrystalline cellulose
Pregelatinised maize starch
Sodium starch glycollate
Colloidal anhydrous silica
Sodium laurilsulfate
Magnesium stearate
Coating:
Polyvinyl alcohol
Titanium dioxide (E 171)
Talc
Soya Lecithin
Xanthan Gum

6.2 Incompatibilities

Not applicable.

6.3 Shelf life

3 years.

6.4 Special precautions for storage

This medicinal product does not require any special storage conditions.

6.5 Nature and contents of container

PVC/PVdC/Alu blister.

Pack sizes:
4, 6, 12, 24,

50, and 100

film-coated

tablets. Not

all pack sizes

may be

marketed.

6.6 Special precautions for disposal of a used medicinal product or waste materials derived from such medicinal product and other handling of the product

No special requirements.

7 MARKETING AUTHORISATION HOLDER

Clonmel Healthcare Ltd.
Waterford Road
Clonmel
Co. Tipperary

8 MARKETING AUTHORISATION NUMBER

PA0126/150/001

9 DATE OF FIRST AUTHORISATION/RENEWAL OF THE AUTHORISATION

Date of first
authorisation:
18 November
2005 Date of
last renewal: 25
January 2010

10 DATE OF REVISION OF THE TEXT

June 2018

Appendix C – Storage of Samples

As part of this study we will collect and store subject's biological samples:

- Plasma collection: EDTA venipuncture tube: 4 x 9 ml
- Serum collection: 2 x 7.5 ml STT or equivalent
- Urine: Specimen collection container (10-60 ml). A minimum of 8 ml of urine
- Stool: fresh stool collected in a clean collection container (10-60mls). A minimum of 10g of stool should be collected.
- Respiratory sample: a sputum sample or nasopharyngeal aspirate placed in viral transport media

These samples will be stored in a secure biobank facility at the UCD Centre for Experimental Host Pathogen Research (CEPHR) for current and future medical research.

A Laboratory requisition form must be completed and sent along with the samples to the Central lab.

Sample(s) will be securely stored for up to 15 years. At this time, the sample(s) will be physically destroyed.

Flow chart for Packaging and Shipment of samples

1. • Call courier to arrange pickup
2. • Ensure that all samples are clearly labelled and any central lab paperwork has been completed
3. • Package samples in bubble wrap pouches provided or wrap in bubble wrap casing
4. • Place absorbent material provided in the bottom of the biohazard bottle and pack samples into the bottle - a 50ml limit applies per bottle.
   • Ensure bottle is clearly labelled with infectious disease sticker
5. • Place the bio-bottle in the outer cardboard package and label clearly with infectious and dry-ice stickers
6. • Once all bottles are packaged, ensure the shipment of dangerous good form is completed.
   • The courier company will have prepared most of this form.
   • Complete the volume and signature/place/date section
7. • Complete courier delivery paperwork
8. • The sample is now ready for collection

Appendix D - Adverse Events and Serious Adverse Events

Definition of an Adverse Event (AE)

An adverse event (AE) is any untoward medical occurrence in a patient administered a medicinal product and which does not necessarily have a causal relationship with this treatment or investigations occurring as part of the study protocol.

An adverse event can therefore be any unfavourable and unintended sign (including an abnormal laboratory finding), symptom or disease temporally associated with the use of a medicinal product, whether or not considered related to this medicinal product. (Definition per International Conference on Harmonization (ICH)).

This includes any occurrence that is new in onset or aggravated in severity or frequency from the baseline condition, or abnormal results of diagnostic procedures, including laboratory test abnormalities.

Adverse Reaction (AR)

Adverse reactions (AR) are all untoward and unintended responses to a medicinal product related to any dose. The phrase 'responses to a medicinal product' means that a causal relationship between a study medication and an AE is at least a reasonable possibility, i.e., the relationship cannot be ruled out.

All cases judged by either the reporting medically qualified professional or the sponsor as having a reasonable suspected causal relationship to the study medication qualify as adverse reactions.

Serious Adverse Event

A serious adverse event as defined by ICH is any untoward medical occurrence that at any dose:

- results in death
- is life-threatening
  (i.e. the patient was at risk of death at the time of the event. It does not refer to an event that hypothetically might have caused death if it were more severe.)
- requires inpatient hospitalisation or prolongation of existing hospitalisation
  (inpatient hospitalisation is defined as hospitalisation for >/= 24 hours.)
- results in persistent or significant disability/incapacity, or
- is a congenital anomaly/birth defect
- any other important medical event
  (Some medical events may jeopardise the subject or may require an intervention to prevent one of the above characteristics/consequences. Such events should also be considered as 'serious' in accordance with the definition.)

Note: Medical and scientific judgment should be exercised in deciding whether expedited reporting is also appropriate in situations other than those listed above. For example, important medical events may not be immediately life threatening or result in death or hospitalisation, but may jeopardise the patient or may require intervention to prevent one of the outcomes listed in the definition above. Any adverse event is considered a serious adverse event if it is associated with clinical signs or symptoms judged by the investigator to have a significant clinical impact.

Clarification on the definition of "severe" and "seriousness"

The term "severe" is often used to describe the intensity (severity) of a specific event (as in mild, moderate or severe myocardial infarction); the event itself, however, may be of relatively minor medical significance (such as severe headache). This is not the same as "serious", which is based on patient/event outcome or action criteria usually associated with events that pose a threat to a patient's life or functioning. Seriousness (not severity) serves as a guide for defining regulatory reporting obligations.

Unexpected Adverse Event

An unexpected adverse event is an adverse event where the nature or severity of which is not consistent with the applicable product information.

For the study drugs involved in this study which have a marketing authorization (commercial agent), the expectedness of an adverse event will be determined by whether or not it is listed in the summary of product characteristics (SPC).

For this study, the current versions of the Hydroxychloroquine and Azithromycin SPCs will be used to assess the expectedness of the event in the study

Suspected Unexpected Serious Adverse Reactions (SUSAR)

A SUSAR is defined as a serious adverse reaction, the nature or severity of which is not consistent with the applicable product information (summary of product characteristics for an authorised medicinal product).

Adverse Events associated With the Use of the IMPs

The investigator's assessment of causality must be made for all AEs (serious and non-serious); the investigator must record the causal relationship in the patients' notes, as appropriate, and report such an assessment in accordance with serious adverse reporting requirements, if applicable.

An adverse event is considered associated with the use of the IMP/treatment if the attribution is definitely, probably or possibly related.

Abnormal Laboratory Assessments:

Abnormal laboratory assessments (including all scanning procedures) determined by the investigator as clinically significant, must be recorded as AEs or SAEs as defined in the above definitions. The investigator should exercise his/her medical and scientific judgment in deciding whether an abnormal laboratory finding, or other abnormal assessment is clinically significant.

Appendix E – Laboratory reference ranges

Test	Abbott Ref Range	MMUH Hospital Ref Range PATHOLOGY LABORATORY October 2019	Comments: Source of References Ranges (2.5-97.5th) is mainly MMUH unless otherwise stated
**Chemistry**			
ALT	0-55 u/L	**0-55 IU/L**	
AST	5-34 U/L	**19-42 IU/L (M<40, F<32)**	
Albumin P	35-50 g/L (34-48 IF >60)	**35-50 g/L <14yr: 38-54, 14-18yr: 32-45, >18yr: 35-52)**	Kit insert
ALK	40-150 U/L (>20Y M, >15Y F)	**30-130 IU/L**	Pathology Harmonization
Amylase	23-96 U/L	**28-97 97 IU/L (Plasma); 0 - 470 IU/L (Urine).**	
Bil Total	3.4-20.5 umol/L	**5-24 µmol/L (M<24, F<15)**	
Bilirubin direct	0-8.6 umol/L (25-66 Y)	**2.4-8.1 µmol/l**	
Chol	NA	**<5.18 mmol/L**	<5.18 is optimal (NCEP guidelines)
Chol HDL	NA	**>1.5 mmol/L**	>1.55 is optimal <1.04 confers CV risk (NCEP guidelines)
Chol LDL	NA (mmol/L)	**<2.6 mmol/L**	<2.60 is optimal (NCEP guidelines)
CK	30-200 (M) + 29-168 (F) U/L	**Ref range for Caucasians 33-208 IU/L (Female), 44-272 (Male).Ref range for Afro-Caribbean twice these**	
	**FEMALE**	**33-208 IU/L**	
	**MALE**	**44-272 IU/L**	
Chloride	101-110 mmol/L	**95-108 mmol/L**	Pathology Harmonization
C02	22-29 mmol/L	**22-29 mmol/L**	Pathology Harmonization (= Abbott)
Creatinine (enz)	64-104 (M), 49-90 (F) umol/L		
	**FEMALE**	**Plasma:46-86 Urine: 5000 – 13000 µmol/L**	
	**MALE**	**Plasma: 65-107 Urine: 7000 – 17000 µmol/L**	
CRP	<5 mg/L	**<7 mg/L**	
GGT	12-64 (M) and 9-36 (F) U/L		
	**FEMALE**	**8-53 IU/L**	
	**MALE**	**11-67 IU/L**	

Glucose	3.89-5.83 mmol/L	**Fasting: 3.5 – 6.0 mmol/L. 2 hour post 75 g load:up to 7.8 mmol/L**.	Use WHO guideline: Normal ≤6.0 mmol/L(Fasting)
Iron (non-fasting)	5.5-25.78 (M), 4.48-27.92 (F) umol/L		
	**FEMALE**	**6-33 µmol/L**	
	**MALE**	**12-32 µmol/L**	
K	3.5-5 mmol/L (wider K phoning range for Dial/HDU/ITU):	**3.5-5.0 mmol/L**	Pathology Harmonization
Lactate	**0.5-2.2 mmol/L**	**0.5-2.0 mmol/L**	Kit insert
LDH	125-220 U/L	**120-220 IU/L**	
Mg	0.66-1.07 mmol/L	**0.70-1.00 mmol/L**	Pathology Harmonization
Na	137-144 mmol/L	**133-146 mmol/L**	Pathology Harmonization
Osmolality (Serum)		**285-295 mOsm/kg**	
Phos	0.74-1.52 mmol/L	**0.74-1.52** mmol/L	Kit insert
Protein (Total)	64-83 g/L (ambulatory)	**65-83 g/L**	Pathology Harmonization
Transferrin	**1.8-3.82** (14-60Y) **+ 1.73-3.60** (60-80Y**) (FEMALE**	**1.93-3.08 g/L**	
	**1.74-3.64** (14-60Y) + **1.63-3.44** (60-80Y) **MALE**	**1.88-3.02 g/L**	
Trig	<1.7 mmol/L = desirible	**<1.7**	<1.7 is optimal (NCEP guidelines)
Urea	2.5-9 mmol/L (incl. both sexes + </> 50y)	**2.8-8.6 mmol/L**	**Plasma: 2.8– 8.6 mmol/L. Urine: 400 – 800 mmol/24hrs**
Uric Acid	210-420 (M) + 150-350 (F) **µmol/L**	**177-465 µmol/L**	**Plasma: 177-465 µmol/L. Urine: 1.2 – 4.0 mmol/L /24hrs**
	(180-385 - av of sexes)		
**Immunoassay:**			
AFP	1.09-8.04 ng/ml	**0-9 µg/L**	
Anti-thyroglobulin	<4.11 IU/ml	**0.45-4.7 IU/mL**	10% CV = 4.11. Use for research but report Immulite and RIA on all samples **Thyroglobulin antibodies are measured on all samples, and if positive, a sample is sent to Birmingham for concurrent measurement of Thyroglobulin to assess interference**.
AntI-TPO	<5.61 IU/ml	**< 5.6 IU/ml**	10% CV = 5.61. Use 5.61
B12	189-883 pg/ml (ng/L)	**211-760 ng/L**	
BETA-HCG	**<5 mIU/ml**	**<5 mIU/ml**	Kit insert
BNP	**<135 ng/L**	**<135 ng/L**	Kit insert
CA-199	**3-37 u/ml (94.4%)**	**2-23 U/mL**	
CA-15-3	**31.3 U/ml (99%F/98.5M)**	**0-31.3 kU/L**	Kit insert
CA-125	**35 U/ml (94%F)**	**0-35 U/mL**	Kit insert
CEA	**<5 ng/ml (93.5%)**	**0-5 µg/L**	Kit insert

CKMB (Mass)	**3.4 AND 7.2 ng/ml (99% F AND M)**	**Total (range) + CKMB % but no range**	**Not tested- hsTnI used instead**
Cortisol	101.2-535.7 nmol/L	**150-455 nmol/L (AM sample)**	No range quoted for random or midnight
Ferritin	**21.81-274.66 (M)** + 4.63-204 ng/ml (f)		*=(ug/L)
	**FEMALE**	**8-247 µg/L**	Females not higher than 200 ug/L HSE guidelines
	**MALE**	**22-275 µg/L**	
Folate	7-31.4 ng/ml	**3.8-18.2 µg/L**	=ug/L
Folate Red Cell	**235.1-788.5 ug/L**	**126-651 µg/L**	Kit insert
FSH	0.95-11.95 mIU/ml (Males)<br>Female:<br>Follicular phase: 3.0-8.1 IU/L<br>Mid-cycle peak: 2.6-16.7 IU/l<br>Luteal phase: 1.4-5.5 IU/l	**1.4-10.8 (M)**	Kit insert quoted for females
freeT4	0.7-1.48 x 12.87 = 9-19 pmol/L	**9-20 pmol/L**	
freeT3	1.71-3.71 pg/ml x 1.536 = 2.6-5.7 pmol/L	**2.6-4.9 pmol/L**	
Insulin	NA (+ not specified)	**NA**	**NA Dependent on plasma glucose concentration**
i-PTH	15-68.3 pg/ml x 0.106 = 1.59-7.24 pmol/L	**1.6-6.9 pmol/L**	
LH	1.14-8.75 mIU/ml (male)<br>Female:<br>Follicular phase: 1.8-11.8 IU/l<br>Mid-cycle peak: 7.6-89.1 IU/l<br>Luteal phase: 0.6-14.0 IU/l	**1.4-6.5 mIU/ml (male)**	Kit insert quoted for females
Oestradiol	**40-161 pmol/L (Males)**	**40-161 pmol/L**	Kit insert
pmol/L	Follicular	**92-921 pmol/L**	Kit insert. Pre-menopausal
	Mid-Cycle	**139-2382 pmol/L**	Kit insert. Pre-menopausal
	Luteal	**92-1145 pmol/L**	Kit insert. Pre-menopausal
Progesterone	NA	**NA**	Revisit RCOG guidelines for source of assay providing diagnostic progesterone concentrations **Assuming correctly timed sample: <10nmol/l ovulation unlikely; 10-25nmol/L equivocal; 25nmol/l: ovulation likely**
Prolactin	3.56-19.4(m) 5.18-26.53 (f) ng/ml X21		
	**Female 109-557 mIU/L**	**Females: 91 - 475 ml/l.**	M: 80-300<br>F: 100-550 HSE guidelines Oct 2019
	**Male 75-407 mIU/L**	**Males**: **73 - 407 mIU/l,**	

SHBG	13.5-71.4 (M) 19.8-155.2 nmol/L (F)	**Male: 11 – 73 nmol/L**<br><br>**Females: 25 – 167 nmol/L (Pre-menopausal: 26.6 – 165.9 nmol/L, Post-menopausal: 20 – 124 nmol/L)**	Established using an earlier cohort of MPH (Healthcheck) samples (Apr/May 2011)
TnI	NA: 0.013/0.033 ug/L f/m = 99% centile	**16 ng/L (F), 34 ng/L (M)**	Use <0.03. 0.03-0.3. Correlate clinically. >0.3 suggests MI in clinically relevant circumstances
Hs_TnI	Range (99th centile) : Male: >34 ng/L (n=766) Female: >16 ng/L (n=764) Critical Value >300 ng/L	Range (99th centile) : Male: >34 ng/L (n=766) Female: >16 ng/L (n=764) Critical Value >300 ng/L	Source of References Ranges (2.5-97.5th) is mainly MMUH unless otherwise stated Interpretation: MI should not be ruled out on the basis of a single negative early Troponin I value. For hsTNI diagnostic guideline please refer to Maternet/ Medical/ Medicine/ Clinical Biochemistry
Total PSA	**M <49 + f = <4 ng/ml then % >4 increase from 2.5 to 7.3 for each decade**	<4<br>Age/PSA:<br>50 – 59 yrs ≥ 3.0 ng/ml<br>60 – 69 yrs ≥ 4.0 ng/ml<br>70 years and over > 5.0 ng/ml	Less than 50 years: < 2.00<br>50 – 59 years: < 3.00<br>60 – 69 years: < 4.00<br>≥ 70 years: < 5.00<br>Source: NCCP Prostate Cancer GP Referral Guideline 2018
TSH	0.35-4.94 uIU/ml	**0.35-4.94 mIU/L**	Kit insert

Appendix F - Sample Drug Labels

The labels noted below (I and II) will be added to the secondary packaging by the site trial pharmacy. Study drug will be kept with the primary packaging at all times.

1. Hydroxychloroquine Label

LABEL I OF II
22 Hydroxychloroquine 200 mg tablets
DAY 1: Take 2 tablets (400 mg) TWICE daily
DAY 2-10: Take one tablet (200 mg) TWICE daily
Store below 30°C
Expiry date: _________ Batch Number: _________
Subject code: _________

2. Azithromycin Label

LABEL I OF II
6 Azithromycin 250 mg tablets
DAY 1: Take 2 tablets (500 mg) ONCE daily
DAY 2-5: Take one tablet ONCE daily
Store below 30°C
Expiry date: _________ Batch Number: _________
Subject code: _________

LABEL II OF II **(common to both)**
For clinical trial use only
Keep out of reach of children
EudraCT: 2020-001265-36
Sponsor: University College Dublin
PIs: Prof P Mallon, Dr E Feeney, Dr A Cotter
University College Dublin School of Medicine, Belfield, Dublin 4, Ireland
Tel: ____________

Appendix G – Declaration of Helsinki

64th WMA General Assembly, Fortaleza, Brazil, October 2013

Ethical Principles for Medical Research Involving Human Subjects

In 1964, the World Medical Association drew up a code of ethics on human experimentation. This code, known as the Declaration of Helsinki, adopted by the 18th WMA General Assembly, Helsinki, Finland, June 1964 and amended by 29th WMA General Assembly, Tokyo, Japan, October 1975, 35th WMA General Assembly, Venice, Italy, October 1983, 41st WMA General Assembly, Hong Kong, September 1989, 48th WMA General Assembly, Somerset West, Republic of South Africa, October 1996, 52nd WMA General Assembly, Edinburgh, Scotland, October 2000, 53rd WMA General Assembly, Washington DC, USA, October 2002 (Note of Clarification added), 55th WMA General Assembly, Tokyo, Japan, October 2004 (Note of Clarification added), 59th WMA General Assembly, Seoul, Republic of Korea, October 2008 and the 64th WMA General Assembly, Fortaleza, Brazil, October 2013 reads:

Preamble

1. The World Medical Association (WMA) has developed the Declaration of Helsinki as a statement of ethical principles for medical research involving human subjects, including research on identifiable human material and data.

The Declaration is intended to be read as a whole and each of its constituent paragraphs should be applied with consideration of all other relevant paragraphs.

2. Consistent with the mandate of the WMA, the Declaration is addressed primarily to physicians. The WMA encourages others who are involved in medical research involving human subjects to adopt these principles.

General Principles

3. The Declaration of Geneva of the WMA binds the physician with the words, “The health of my patient will be my first consideration,” and the International Code of Medical Ethics declares that, “A physician shall act in the patient's best interest when providing medical care.”

4. It is the duty of the physician to promote and safeguard the health, well-being and rights of patients, including those who are involved in medical research. The physician's knowledge and conscience are dedicated to the fulfilment of this duty.

5. Medical progress is based on research that ultimately must include studies involving human subjects.

6. The primary purpose of medical research involving human subjects is to understand the causes, development and effects of diseases and improve preventive, diagnostic and therapeutic interventions (methods, procedures and treatments). Even the best proven interventions must be evaluated continually through research for their safety, effectiveness, efficiency, accessibility and quality.

7. Medical research is subject to ethical standards that promote and ensure respect for all human subjects and protect their health and rights.

8. While the primary purpose of medical research is to generate new knowledge, this goal can never take precedence over the rights and interests of individual research subjects.

9. It is the duty of physicians who are involved in medical research to protect the life, health, dignity, integrity, right to self-determination, privacy, and confidentiality of personal information of research subjects. The responsibility for the protection of research subjects must always rest with the physician or other health care professionals and never with the research subjects, even though they have given consent.

10. Physicians must consider the ethical, legal and regulatory norms and standards for research involving human subjects in their own countries as well as applicable international norms and standards. No national or international ethical, legal or regulatory requirement should reduce or eliminate any of the protections for research subjects set forth in this Declaration.

11. Medical research should be conducted in a manner that minimises possible harm to the environment.

12. Medical research involving human subjects must be conducted only by individuals with the appropriate ethics and scientific education, training and qualifications. Research on patients or healthy volunteers requires the supervision of a competent and appropriately qualified physician or other health care professional.

13. Groups that are underrepresented in medical research should be provided appropriate access to participation in research.

14. Physicians who combine medical research with medical care should involve their patients in research only to the extent that this is justified by its potential preventive, diagnostic or therapeutic value and if the physician has good reason to believe that participation in the research study will not adversely affect the health of the patients who serve as research subjects.

15. Appropriate compensation and treatment for subjects who are harmed as a result of participating in research must be ensured.

Risks, Burdens and Benefits

16. In medical practice and in medical research, most interventions involve risks and burdens.

Medical research involving human subjects may only be conducted if the importance of the objective outweighs the risks and burdens to the research subjects.

17. All medical research involving human subjects must be preceded by careful assessment of predictable risks and burdens to the individuals and groups involved in the research in comparison with foreseeable benefits to them and to other individuals or groups affected by the condition under investigation.

Measures to minimise the risks must be implemented. The risks must be continuously monitored, assessed and documented by the researcher.

18. Physicians may not be involved in a research study involving human subjects unless they are confident that the risks have been adequately assessed and can be satisfactorily managed.

When the risks are found to outweigh the potential benefits or when there is conclusive proof of definitive outcomes, physicians must assess whether to continue, modify or immediately stop the study.

Vulnerable Groups and Individuals

19. Some groups and individuals are particularly vulnerable and may have an increased likelihood of being wronged or of incurring additional harm.

All vulnerable groups and individuals should receive specifically considered protection.

20. Medical research with a vulnerable group is only justified if the research is responsive to the health needs or priorities of this group and the research cannot be carried out in a non-vulnerable group. In addition, this group should stand to benefit from the knowledge, practices or interventions that result from the research.

Scientific Requirements and Research Protocols

21. Medical research involving human subjects must conform to generally accepted scientific principles, be based on a thorough knowledge of the scientific literature, other relevant sources of information, and adequate laboratory and, as appropriate, animal experimentation. The welfare of animals used for research must be respected.

22. The design and performance of each research study involving human subjects must be clearly described and justified in a research protocol.

The protocol should contain a statement of the ethical considerations involved and should indicate how the principles in this Declaration have been addressed. The protocol should include information regarding funding, sponsors, institutional affiliations, potential conflicts of interest, incentives for subjects and information regarding provisions for treating and/or compensating subjects who are harmed as a consequence of participation in the research study.

In clinical trials, the protocol must also describe appropriate arrangements for post-trial provisions.

Research Ethics Committees

23. The research protocol must be submitted for consideration, comment, guidance and approval to the concerned research ethics committee before the study begins. This committee must be transparent in its functioning, must be independent of the researcher, the sponsor and any other undue influence and must be duly qualified. It must take into consideration the laws and regulations of the country or countries in which the research is to be performed as well as applicable international norms and standards but these must not be allowed to reduce or eliminate any of the protections for research subjects set forth in this Declaration.

The committee must have the right to monitor ongoing studies. The researcher must provide monitoring information to the committee, especially information about any serious adverse events. No amendment to the protocol may be made without consideration and approval by the committee. After the end of the study, the researchers must submit a final report to the committee containing a summary of the study's findings and conclusions.

Privacy and Confidentiality

24. Every precaution must be taken to protect the privacy of research subjects and the confidentiality of their personal information.

Informed Consent

25. Participation by individuals capable of giving informed consent as subjects in medical research must be voluntary. Although it may be appropriate to consult family members or community leaders, no individual capable of giving informed consent may be enrolled in a research study unless he or she freely agrees.

26. In medical research involving human subjects capable of giving informed consent, each potential subject must be adequately informed of the aims, methods, sources of funding, any possible conflicts of interest, institutional affiliations of the researcher, the anticipated benefits and potential risks of the study and the discomfort it may entail, post-study provisions and any other relevant aspects of the study. The potential subject must be informed of the right to refuse to participate in the study or to withdraw consent to participate at any time without reprisal. Special attention should be given to the specific information needs of individual potential subjects as well as to the methods used to deliver the information.

After ensuring that the potential subject has understood the information, the physician or another appropriately qualified individual must then seek the potential subject's freely-given informed consent, preferably in writing. If the consent cannot be expressed in writing, the non-written consent must be formally documented and witnessed.

All medical research subjects should be given the option of being informed about the general outcome and results of the study.

27. When seeking informed consent for participation in a research study the physician must be particularly cautious if the potential subject is in a dependent relationship with the physician or may consent under duress. In such situations the informed consent must be sought by an appropriately qualified individual who is completely independent of this relationship.

28. For a potential research subject who is incapable of giving informed consent, the physician must seek informed consent from the legally authorised representative. These individuals must not be included in a research study that has no likelihood of benefit for them unless it is intended to promote the health of the group represented by the potential subject, the research cannot instead be performed with persons capable of providing informed consent, and the research entails only minimal risk and minimal burden.

29. When a potential research subject who is deemed incapable of giving informed consent is able to give assent to decisions about participation in research, the physician must seek that assent in addition to the consent of the legally authorised representative. The potential subject's dissent should be respected.

30. Research involving subjects who are physically or mentally incapable of giving consent, for example, unconscious patients, may be done only if the physical or mental condition that prevents giving informed consent is a necessary characteristic of the research group. In such circumstances the physician must seek informed consent from the legally authorised representative. If no such representative is available and if the research cannot be delayed, the study may proceed without informed consent provided that the specific reasons for involving

subjects with a condition that renders them unable to give informed consent have been stated in the research protocol and the study has been approved by a research ethics committee. Consent to remain in the research must be obtained as soon as possible from the subject or a legally authorised representative.

31. The physician must fully inform the patient which aspects of their care are related to the research. The refusal of a patient to participate in a study or the patient's decision to withdraw from the study must never adversely affect the patient-physician relationship.

32. For medical research using identifiable human material or data, such as research on material or data contained in biobanks or similar repositories, physicians must seek informed consent for its collection, storage and/or reuse. There may be exceptional situations where consent would be impossible or impracticable to obtain for such research. In such situations the research may be done only after consideration and approval of a research ethics committee.

Use of Placebo

33. The benefits, risks, burdens and effectiveness of a new intervention must be tested against those of the best proven intervention(s), except in the following circumstances:

Where no proven intervention exists, the use of placebo, or no intervention, is acceptable; or

Where for compelling and scientifically sound methodological reasons the use of any intervention less effective than the best proven one, the use of placebo, or no intervention is necessary to determine the efficacy or safety of an intervention

and the patients who receive any intervention less effective than the best proven one, placebo, or no intervention will not be subject to additional risks of serious or irreversible harm as a result of not receiving the best proven intervention.

Extreme care must be taken to avoid abuse of this option.

Post-Trial Provisions

34. In advance of a clinical trial, sponsors, researchers and host country governments should make provisions for post-trial access for all participants who still need an intervention identified as beneficial in the trial. This information must also be disclosed to participants during the informed consent process.

Research Registration and Publication and Dissemination of Results

35. Every research study involving human subjects must be registered in a publicly accessible database before recruitment of the first subject.

36. Researchers, authors, sponsors, editors and publishers all have ethical obligations with regard to the publication and dissemination of the results of research. Researchers have a duty to make publicly available the results of their research on human subjects and are accountable for the completeness and accuracy of their reports. All parties should adhere to accepted guidelines for ethical reporting. Negative and inconclusive as well as positive results must be published or otherwise made publicly available. Sources of funding, institutional affiliations and conflicts of interest must be declared in the publication. Reports of research not in accordance with the principles of this Declaration should not be accepted for publication.

Unproven Interventions in Clinical Practice

37. In the treatment of an individual patient, where proven interventions do not exist or other known interventions have been ineffective, the physician, after seeking expert advice, with informed consent from the patient or a legally authorised representative, may use an unproven intervention if in the physician's judgement it offers hope of saving life, re-establishing health or alleviating suffering. This intervention should subsequently be made the object of research, designed to evaluate its safety and efficacy. In all cases, new information must be recorded and, where appropriate, made publicly available.
