# Supplementary material for: The COVIRL-001 Trial: A multicentre, prospective, randomised trial comparing standard of care (SOC) alone, SOC plus hydroxychloroquine monotherapy or SOC plus a combination of hydroxychloroquine and azithromycin in the treatment of non- critical, SARS-CoV-2 PCR-positive population not requiring immediate resuscitation or ventilation but who have evidence of clinical decline: A structured summary of a study protocol for a randomised controlled trial
Source: Trials. 2020 May 25;21:430. doi: 10.1186/s13063-020-04407-x (PMC7247433; doi:10.1186/s13063-020-04407-x)
Supplement: Supplementary file 3 — Additional file 3. COVIRL_PIL. [file 13063_2020_4407_MOESM3_ESM.pdf]

PATIENT INFORMATION STATEMENT AND CONSENT FORM

COVID-19 Pre-ICU Trials Ireland
Treatment Study 001

COVIRL-001 - A multicentre, prospective, randomised trial comparing standard of care (SOC) alone, SOC plus hydroxychloroquine monotherapy or SOC plus a combination of hydroxychloroquine and azithromycin in the treatment of non-critical, SARS-CoV-2 PCR-positive population not requiring immediate resuscitation or ventilation but who have evidence of clinical decline.

Version 1.1 dated 18/04/2020

Study sponsor: University College Dublin

Sponsor’s Protocol Code: UCDCRC/20/01

Primary Funder: University College Dublin

EudraCT number: 2020-001265-36

[INSERT STUDY SITE HERE]

Chief Investigator: Prof. Patrick Mallon
Professor of Microbial Diseases
Centre for Experimental Pathogen Host Research (CEPHR)
UCD School of Medicine
Consultant Infectious Diseases Physician
St Vincent’s University Hospital

Site Principal Investigator: [Insert details]

Data Controller (s)

Name:	University College Dublin
Registered office at:	Belfield, Dublin 4
Company Type:	Education
Registration number:	239961
Name:	St Vincent's University Hospital
Registered office at:	Elm park, Dublin 4
Company Type:	Healthcare
Registration number:	338585
Name:	Mater Misericordiae University Hospital
Registered office at:	Eccles Street, Dublin 7
Company Type:	Healthcare
Registration number:	351402

Data Protection Officer (s)

St Vincent's University Hospital
Orlaith McCarthy, SVUH, Elm Park, Dublin 4
Email: dataprotection@svuh.ie
Telephone: 01 221 3591

University College Dublin
Ulrike Kolch, Roebuck Castle, Belfield, Dublin 4
Email: gdpr@ucd.ie

Mater Misericordiae University Hospital
Ronan O'Halloran, MMUH, Eccles St, Dublin 7
Email: dpo@mater.ie
Telephone: 01 803 4035

1. Introduction

We invite you to take part in a research study about COVID-19. It is up to you whether or not you want to join this study. Please take time to read this information and ask the staff any questions you may have before agreeing to take part. You can take as much time as you need to decide.

2. Why are we conducting this research?

This study aims to compare how effective two drugs are in treating people infected with COVID-19 who are in hospital and whose breathing problems are getting worse. These two drugs are currently approved for treatment of conditions other than COVID-19.

COVID-19, often referred to as “coronavirus” is a disease which was first reported in China in late 2019. It has since spread worldwide, and has been declared a pandemic by the World Health Organisation.

COVID-19 is highly infectious, spreading quickly within populations around the world. Many people with COVID-19 get serious symptoms, meaning they are admitted to hospital. Patients with very serious infections have to be admitted to the Intensive Care Unit (ICU). COVID-19 has become a serious public health concern. It is hoped that having effective treatments would lessen the burden on our health systems while dealing with this pandemic, and would result in better outcomes for patients infected.

So far, an effective treatment for COVID-19 has not been found. However, several drugs are being tested in clinical trials internationally, and two of these drugs are hydroxychloroquine and azithromycin. We will be testing these two drugs in this study.

Hydroxychloroquine was first developed to treat malaria, but is now more commonly used to treat inflammation in autoimmune conditions such as rheumatoid arthritis.

Azithromycin is an antibiotic used for treatment of a number of bacterial infections.

3. The Study Design

We aim to enrol 267 people with COVID-19 who have been hospitalised, and whose breathing problems are getting worse but can breathe on their own.

The study is ‘open-label’: this means both the researchers and participants know which treatment the participant gets. It is a three-arm study: this means there are three treatment options. The study is also randomised: this means participants have an equal chance of being put into one of the following three treatment groups:

- The group getting standard of care (a third of those who join)

- The group getting hydroxychloroquine for 10 days (a third of those who join)

- The group getting hydroxychloroquine for 10 days and azithromycin for 5 days (a third of those who join)

The treatment you get will been chosen at random by a computer. Your doctor has no influence on the treatment chosen for you. You will have a 33% (1 in 3) chance of getting any of the 3 options.

Taking part this study will not change any other treatments that your doctor feels you need in the management of your infection. You may be asked to take part in other clinical trials even though you are on this study.

4. Clinic Visits and Procedures

The study will involve a range of assessments and procedures over the course of 2 months during and/or after your admission to hospital.

If you are a woman, you cannot join if you are pregnant or breastfeeding. A pregnancy test will be done before you are enrolled in the study. We will also do a second test at day 5 to make sure you are not pregnant while on study.

The study procedures will be the same for all three study groups.

Please see below a description of the procedures included in the study visits:

Visit 1: Screening visit:

At the screening visit, the study doctor or nurse will invite you to take part in the study if you are eligible. If you agree to take part, they will ask you to sign a consent form. A copy of the consent form will be given to you if it is safe to do so during your period of isolation. If it is not safe to do so immediately, we will give you a copy once you have come out of isolation. After signing the consent form, the study doctor/nurse will:

- Collect information about your health and medication history, including when you first experienced symptoms of COVID-19 infection
- Check your health status by doing a full physical examination
- Check your heart and lung function by doing an ECG and a chest X-ray
- Take a nasopharyngeal swab test (a sample from the back of your nose and throat) for COVID-19 (unless a previous result is available)
- In women of child-bearing age, perform a pregnancy testing (blood or urine) to make sure you are not pregnant prior to taking part in the study.

You will then be given a study number and this will be used in recording all study information about you.

Visit 2: Baseline visit

Copies of the screening visit forms will be reviewed and some details may be checked again with you.

This visit will involve a brief physical examination and blood tests.

You will be asked to give an extra blood sample as well as urine, stool and respiratory samples (a swab from the back of your nose or throat, or a sputum sample). These sample will be stored and will be used to learn more about COVID-19.

At this visit you will be randomly allocated (like tossing a coin) to one of the 3 study groups and study medication will be prescribed if relevant to your treatment group.

The study medication will consist of either hydroxychloroquine for 10 days, or both hydroxychloroquine for 10 days and azithromycin for 5 days.

Visits from baseline to day 60:

Subsequent visits will take place at day 2, 3, 5, 10, 14, 21, 28 and 60 and will involve:

- clinical and medical assessment, review of medications and side effects or new conditions that may have arisen since the previous visit

- physical examination and blood tests to check your general health
- ECG (day 3 and day 10)
- Chest X-ray (only where needed after day 10 visit)
- Blood, urine and stool samples for storage (day 5, 10, 28 and 60)
- Respiratory samples (day 3, 5, 10, 14 and 28)

Note: Study procedures will be done while you are still in hospital and can be done after you are discharged.

5. Study medications

The study medication is either:

- **hydroxychloroquine 200 mg oral tablets:** 2 tablets twice a day on day 1 followed by 1 tablet twice daily for day 2 to 10.
- both **hydroxychloroquine as described above** and **azithromycin 250 mg oral tablets:** 2 tablets once a day on day 1 followed by 1 tablet once daily from day 2 to 5

How to take these tablets:

- Hydroxychloroquine should be taken with a meal or glass of milk
- Azithromycin should be taken at least one hour before or 2 hours after food.

Interaction with other medications

Some medications can affect how hydroxychloroquine or azithromycin work and may affect their effectiveness or increase their side effects. This is why we will check what medications you are taking before you take part in the study and will update your medical records during the study.

Risks related to the study medication

Hydroxychloroquine

Hydroxychloroquine can cause changes to the rhythm of your heartbeat and so we will do regular ECGs to make sure this is not happening. We will also check your blood test results for the side effects of hydroxychloroquine.

Use with caution in:

- patients with kidney / liver impairment and those taking medicines known to affect those organs
- patients with severe gastrointestinal, neurological or blood disorders
- patients with a sensitivity to quinine, those with glucose-6-phosphate dehydrogenase deficiency, those with porphyria cutanea tarda.

The study doctor or nurse will review your medical history at screening visit prior to enrolment in the study for any evidence of such disorders.

Side effects:

This is a list of the most common side effects of hydroxychloroquine:

- Very common side effects (more than 1 in 10 people): abdominal (tummy) pain, nausea (feeling sick)
- Common side effects (up to 1 in 10 people): diarrhoea, vomiting, loss of appetite, change in mood, headache, blurring of vision, skin rash, itchy skin

Azithromycin

- Azithromycin can also cause changes to the rhythm of your heartbeat and so we will do regular ECGs to make sure this is not happening.

Use with caution in:

- patients with kidney/liver impairment and those taking medicines known to affect those organs
- patients with myasthenia gravis

The study doctor/nurse will review your medical history at screening visit prior to enrolment in the study for any evidence of such disorders.

Side effects:

Here there is a list of the most common side effects of azithromycin:

- Very common side effects (observed in more than 1 in 10 people): diarrhoea, abdominal (tummy) pain, nausea (feeling sick), flatulence (wind)
- Common side effects (up to 1 in 10 people): vomiting, indigestion, loss of appetite, changes in sense of taste, headache, dizziness, somnolence (feeling sleepy), tingling, problems with eyesight, deafness, rash, itchy skin, joint pain, fatigue, abnormal white blood cells counts

6. Risks of Study Procedures

As part of this study, you will have your blood drawn (about 60 mls or 12 teaspoons) at each visit. This procedure is uncomfortable but rarely results in any significant problems. Side-effects that have been noted with drawing blood include feeling light-headed or faint, fainting, formation of a blood clot, bruising and/or infection at the site of the needlestick.

If you take part in this study you will have chest x-rays. Some of these will be extra to those that you would have if you did not take part. These procedures use ionising radiation to form images of your lungs. Ionising radiation can cause cell damage that may, after many years or decades, turn cancerous. We are all at risk of developing cancer during our lifetime. The normal risk is that this will happen to about 50% of people at some point in their life. Taking part in this study will add only a very small chance of this happening to you.

If you are a woman of childbearing potential you must not be pregnant if you wish to take part in the study. A pregnancy test will be taken at screening visit and again during the study to ensure that you are eligible to take part.

7. Sample storage

As part of this study we will collect and store blood, urine, stool, and respiratory samples from study visits. These samples will be stored in a secure biobank facility at the UCD Centre for Experimental Host Pathogen Research (CEPHR) for current and future medical research that relates to the treatment of COVID-19 and the immune system.

If you chose to consent to it, we will study your genetics as well as the genetics of the COVID-19 virus. We will also study your response to the treatments being tested in this study and how your body's immune system and blood system respond to the infection and the treatments. When we do this research, we will approach the ethics committees again for further approval prior to doing any studies related to your genetics but we will not ask for additional consent from you provided the ethics committee approves the analyses.

Your sample(s) will not be directly identifiable as your sample. The stored tube(s) will only contain your study code that does not include any of your personal details. Only qualified and authorised laboratory staff will be able to access your stored samples.

Your sample(s) will be securely stored, for up to 15 years. At this time, your sample(s) will be physically destroyed.

8. Benefits of Participating in the Study

You will be part of a study that aims to improve how people with COVID-19 are looked after. You will not benefit directly from taking part in this trial but the information we will obtain may provide further knowledge of COVID-19 and how it may be treated into the future.

9. Alternative Treatments/Procedures

You do not have to take part in this study if you don't want to. Treatments prescribed as part of this study will be in addition to routine standard care, which you will still receive as normal, as your doctors feel is necessary.

10. Compensation

You will not be paid for taking part in this study.

You will not be charged for taking part in this study.

Every effort will be made to prevent any injury that could result from your taking part in this study. However, if you suffer any adverse experience resulting directly from the study medication of a study-related procedure, compensation will be given for the reasonable costs of medical treatment to the extent that such costs are not covered by your medical insurance or government health schemes.

11. Ethical Approval of the study

The [insert IRB details] reviewed and approved this study.

12. Financial Support for the Study

University College Dublin is covering the costs of carrying out this study. University College Dublin is the study sponsor.

13. Questions about the study

You may ask the doctor at the study site if you have questions about the study. He/she will do all that is possible to answer your questions and concerns as they arise. You can also contact him/her by phoning:

If new information becomes available during the course of the study that is relevant to your willingness to continue taking part in the study we will tell you promptly.

If you have a problem during the study and would like to talk to an independent person you [Insert Contact]

14. What happens when the Clinical Trial stops?

This study is exploring the treatment of COVID-19 using medication that is licensed and approved by the EMA (European Medicines Agency) for the treatment of conditions other than COVID-19. When the study has finished your study doctor will inform you of the steps to follow as part of your

normal standard of care. Your study doctor will inform you if other treatment options become available during your admission and advise on whether they are suitable for you. In addition, by participating in this clinical trial you may also still participate in other clinical studies and clinical trials provided you meet the criteria set out by those studies.

15. Statements of Subject Rights

Your participation in this study is entirely voluntary. Your doctor will have explained the details of this study to you and answered any questions you may have. You should be satisfied with the information you have been given and have had enough time to consider whether you want to take part.

If you decide you would like to take part in this study, you will be asked to sign a consent form and you will be given a copy of the signed form to keep.

If you decide not to take part or to withdraw from the study, you can do so at any time without having to give a reason, and you will not be penalised nor lose any benefits to which you are otherwise entitled. However, data collected during your time on the study will still be used. If you wish, any sample that can still be identified as yours will be destroyed. However, information from your sample(s) prior to receiving your request for destruction will not be deleted. If you ask for your sample(s) to be destroyed, you will not lose any benefits, medical treatment or legal rights to which you are otherwise entitled.

If you decide you want to stop taking part in the study, tell the study staff as soon as you can.

- We will tell you how to stop participating in the study.
- If you decide to stop, you can continue getting care from your regular doctor.

In addition, if you switch your treatment during the study we would still like you to continue in the study. The results collected during the study will still be used.

Even if you want to stay in the study, there may be reasons why we will need to withdraw you form the study. If at any time your study doctor considers that it is in your best interest to be withdrawn from the study, he or she will explain the reasons and arrange for your care to continue. The sponsor may also decide to stop the study. If this happens, we will let you know the reason for withdrawing you from the study.

16. Confidentiality and disclosure of information

During this study, your Study Doctor, nurses and other study personnel will collect and record information about you, your health including your ethnic origin, and your participation in the study (known as “Personal Information”) on forms provided by Sponsor. These forms are known as case report forms. To ensure that your Personal Information is kept confidential, your name and any other information that allows you to be identified directly will not be entered on the case report forms or included in any records or samples your doctor provides to the Sponsor or Sponsor’s authorized representatives. Instead, you will only be identified by a code. You will not be identified in any publication or public presentation of data from this study.

During this study we may conduct genetic analyses on your blood samples. The results from these analyses will be used for research purposes only. If we share this information with other researchers, it will only be where the data is fully anonymous; i.e. the data cannot be traced back to you. We will not permit access to any genetic data linked to your identity by third parties.

The Sponsor, parties working with or on behalf of the Sponsor, the Ethics Committee responsible for approving the conduct of the study, local Regulatory Authorities may access your medical records to make sure the study has been done properly and may be able to identify you. Your medical records will be reviewed only at the study centre.

17. Data protection

The **[study site]** and the study sponsor, University College Dublin, and its authorised representatives are committed to protecting and respecting your privacy. This Participant Information and Consent Form together sets out the basis on which any personal data we collect from you or that you provide to us will be processed by us an independent data controller. Please read this Participant Information and Consent Form carefully to understand our treatment and use of your personal data.

The processing of your personal data will be in compliance with the Data Protection Acts 1988 to 2018 (as amended) and the General Data Protection Regulation (the "Data Protection Legislation").

Please note that agreeing to take part in a research program with **[study site]** and University College Dublin, you acknowledge that you have read, understood and agree to this Participant Information and Consent Form.

17.1 Processing of your personal data, including health data

The Personal Information collected about you will be held by **[study site],** the Sponsor (UCD) and Sponsor's authorized representatives, which together are responsible for processing your Personal Information in accordance with applicable data protection laws.

Personal data will be processed mainly for the following purposes on the basis of your consent:

Personal data	Purpose of processing[1]
Identification (name, date of birth, medical record number, racial and ethnic origin (please note this and subsequent information will be anonymised/coded for data processing).	(a) Originally captured as part of medical care<br>(b) used for purpose of carrying out research
Laboratory test results	Clinical care, safety measures, research outcomes
Clinical and or medication history (not identifiable)	(a) Relevant for understanding study outcomes<br>(b) Captured as research outcomes
Questionnaires	(a) Patient reported outcome measures required to assess response to treatment<br>(b) Captured as research outcomes
Diagnostic tools/machines	Captured as research outcomes

[1] *Please list the Personal Data that will be processed and the purpose of processing such Personal Data.*

In addition, the Sponsor and its authorized representatives will analyse and use the Personal Information for purposes which may include:

- checking your suitability to take part in the study,
- monitoring your treatment with the study drug
- comparing and pooling your treatment results with those of other subjects in clinical studies
- establishing whether the study drug meets the appropriate standards of safety set by the authorities,
- establishing whether the study drug is effective,
- supporting the development of the study drug,
- supporting the licensing application for regulatory approval of the study drug anywhere in the world,
- supporting the marketing, distribution, sale and use of the study drug anywhere in the world,
- anonymizing the data to provide to third parties,
- complying with specific regulations governing clinical trials, and/or
- as otherwise required or authorized by law.

Most of the personal data we process is obtained from you directly, but we also obtain personal data about you from your;

- Hospital medical charts/notes,
- laboratory test results,
- hospital pharmacy records
- diagnostic tool such as the chest X-ray and ECG

17.2 Sharing of personal data

Your personal data may in particular be shared with our collaborators listed at the following website link: **http://www.ucd.ie/medicine/cephr/collaborators/**

*NOTE: These parties will either be acting as Processors of your information as part of this research study e.g. clinical research organizations (CROs), non**-[study site]** employees supporting research process or Controllers in their own right.

(i) Service Providers

We will check any third party that we use to ensure that they can provide sufficient guarantees regarding the confidentiality and security of your personal data. We will have written contracts with them which provide assurances regarding the protections that they will give to your personal data and their compliance with our data security standards and international transfer restrictions.

(ii) Disclosures to Third Parties

In certain circumstances, we share and/or are obliged to share your personal data with third parties outside **[study site]** and University College Dublin, for the purposes described above and in accordance with Data Protection Legislation.

These third parties include but are not limited to:

- the Health Products Regulatory Authority;
- the Health Service Executive;
- the Joint Commission International;
- relevant industry bodies;
- external professional advisors; and
- others, where it is permitted by law, or where we have your consent.

17.3 Transfers outside the European Economic Area

Your personal information may be transferred, stored and processed in one or more countries outside the European Economic Area ("EEA"), for example with collaborators or when one of our service providers use employees or equipment based outside the EEA. For transfers of your personal data to third parties outside of the EEA, we take additional steps in line with Data Protection Legislation. We have put in place adequate safeguards with respect to the protection of your privacy, fundamental rights and freedoms, and the exercise of your rights, e.g. we establish an adequate level of data protection through EU Standard Contractual Clauses based on the EU commission's model clauses.

If you would like to see a copy of any relevant provisions, please contact your data protection officer (see "page 2).

17.4 How is my personal data secured?

The **[study site]** and University College Dublin operates and uses appropriate technical and physical security measures to protect your personal data.

We have in particular taken appropriate security measures to protect your personal data from accidental or unlawful destruction, loss, alteration, unauthorised disclosure of, or access, in connection with this research study. Access is only granted on a need-to-know basis to those people whose roles require them to process your personal data. In addition, our service providers are also selected carefully and required to use appropriate protective measures.

17.5 Storage of Personal Data

We will keep your personal data for 15 after the study ends. This may mean that some information is held for longer than other information.

17.6 Your rights

You may have various rights under Data Protection Legislation. However, in certain circumstances, these rights may be restricted[2]. In particular, your rights may be restricted where this is necessary: (i) for the prevention, detection, investigation and prosecution of criminal offences; (ii) in contemplation of or for the establishment, exercise or defence of a legal claim or legal proceedings (whether before a court, tribunal, statutory body or an administrative or out-of-court procedure); and/or (iii) for the performance of a task carried out in the public interest or in the exercise of official authority vested in the **[study site]** and University College Dublin.

These rights may include (as relevant):

(i) The **right of access** enables you to check what type of personal data we hold about you and what we do with that personal data and to receive a copy of this personal data;

(ii) The **right to object** to processing of your personal data where that processing is carried out on the basis of our legitimate interests. We will stop using your personal data unless we can demonstrate an overriding legitimate ground for the continued processing of this personal data;

(iii) The **right to rectification** enables you to correct any inaccurate or incomplete personal data that we hold about you;

(iv) The **right to erasure** enables you to request that we erase personal data held about you in certain circumstances;

(v) The **right to restrict processing** of your personal data by us in certain cases, including if you believe that the personal data held about you is inaccurate or our use of the personal data is unlawful; and

---

[2] *Article 23 of the General Data Protection Regulation, which is transposed into Irish law by section 59 of the Data Protection Act 2018, sets out the circumstances in which your rights may be restricted.*

(vi) The **right to data portability** enables you to receive your personal data in a structured, commonly used and machine readable format and to have that personal data transmitted to another data controller

17.7 Your right to lodge a complaint with a supervisory authority

Without prejudice to any other administrative or judicial remedy you might have, you may have the right under data protection legislation in your country (where applicable) to lodge a complaint with the relevant data protection supervisory authority in your country (i.e. the Office of the Data Protection Commissioner in Ireland) if you consider that we have infringed applicable data protection legislation when processing your personal data. This means the country where you are habitually resident, where you work or where the alleged infringement took place

Version 1.1 dated 18th April 2020

COVID-19 Pre-ICU Trials Ireland
Treatment Study 001

CONSENT FORM

Participants name: ……………………………………………………………

Name of Investigator: …………………………………………………………… **Please initial box**

1.	I confirm that I have read and understand the information sheet (version ___ dated DD / MMM / YYYY) for the above study. I have had the opportunity to consider the information, ask questions and have had these answered satisfactorily.	INITIALS
2.	I understand that my participation is voluntary and that I am free to withdraw at any time without giving any reason, without my medical care or legal rights being affected.	INITIALS
3.	I understand that relevant sections of my medical notes and data collected during the study may be looked at by responsible individuals from the sponsor, hospital or regulatory authorities where it is relevant to my taking part in this research. I give permission for these individuals to access my records.	INITIALS
4.	I give informed explicit consent to have my data processed, including information about my racial and ethnic origin as part of this research.	INITIALS
5.	I agree that my anonymized personal data generated during this study may be transferred, stored and processed in one or more countries outside of the European Economic Area.	INITIALS
6.	I agree for my biological samples be stored for analysis in future ethically approved research projects related to current study, including genetic testing of my genes as well as the genetics of the virus, without further consent being required.	INITIALS
7.	I agree to my GP being informed of my participation in this study and for my GP to disclose information about my medical status to the study doctor.	INITIALS
8.	I consent to be contacted by the study team as part of this research study.	INITIALS
9.	I understand that I will be given a signed copy of this document to keep.	INITIALS
10.	I consent to take part in this research study having been fully informed of the risks, benefits and alternatives.	INITIALS

______________________	______________________	______________
Signature of participant	Please PRINT name	Date/Time
Signature of Person obtaining consent	Please PRINT name	Date/Time
Signature of witness	Please PRINT name	Date/Time

Nature of Witness ____

Version 1.1 dated 18th April 2020

REVOCATION OF CONSENT

I hereby wish to **WITHDRAW** my consent to participate in the research proposal described above and understand that such withdrawal **WILL NOT** jeopardize any treatment or my relationship with the **[study site]**

____________________________ ______________________________ ________________

Signature of participant Please PRINT name Date/Time

The section for Revocation of Consent should be forwarded to:

[Add Principal Investigator Details]
